# Carbon‐Coated Probiotics Restore Intestinal Homeostasis to Promote Damage Repair in Inflammatory Bowel Disease

**DOI:** 10.1002/advs.202509812

**Published:** 2025-07-12

**Authors:** Xue Chen, Haiyan Guo, Yuhan Li, Qiaowen Lin, Hua Liu, Liwen Hong, Lei Wang, Jie Zhong, Dalong Ni, Zhengting Wang

**Affiliations:** ^1^ Department of Gastroenterology Ruijin Hospital Shanghai Jiao Tong University School of Medicine No. 197, Ruijin 2nd Rd Shanghai 200025 P. R. China; ^2^ Department of Orthopaedics Shanghai Key Laboratory for Prevention and Treatment of Bone and Joint Diseases Shanghai Institute of Traumatology and Orthopaedics Ruijin Hospital Shanghai Jiao Tong University School of Medicine No. 197, Ruijin 2nd Rd Shanghai 200025 P. R. China; ^3^ Department of Geriatrics Ruijin Hospital Shanghai Jiao Tong University School of Medicine 197 Ruijin 2nd Road Shanghai 200025 P. R. China

**Keywords:** Carbon dots, Inflammatory bowel disease, Injury healing, Probiotics

## Abstract

Inflammatory bowel disease (IBD) is regarded as green cancer that seriously endangers human quality of life. Orally administered probiotic treatment has been regarded as a promising strategy for the adjunctive treatment of IBD. However, its efficacy is greatly inhibited due to the harsh gut microenvironment and low targeted delivery volume. Herein, carbon dots are applied to modify *Lactobacillus rhamnosus* GG (C dots@LGG) to enhance the anti‐inflammatory and injury healing effects of probiotics. The C‐dots coating bestowed probiotics with a remarkable antioxidant capability, thereby mitigating inflammation and safeguarding LGG from the rigors of gastrointestinal circumstances and oxidative harm within the inflamed colon, as well as facilitating its adhesion and colonization. Conjointly, C dots@LGG reconstructs the intestinal barrier by spurring the proliferation of intestinal epithelial cells, especially goblet cells, and enhancing the intercellular tight junction (TJ). Moreover, C dots@LGG with enhanced metabolic capacity regulated the gut microbiota by enriching its diversity, suppressing the multiplication of harmful bacteria, and fostering that of beneficial bacteria. Collectively, these combined effects endow C dots@LGG to effectively attenuate mice colitis and promote injury healing in vivo. Given its simple synthesis protocol, it is anticipated that C dots@LGG can provide an effective approach for the collaborative non‐immunosuppressive therapy for IBD.

## Graphical abstract

A carbon dots‐coated probiotics hybrid system (C dots@LGG) exerts multi‐functional effects in treating colitis, including scavenging ROS to reduce oxidative damage, modulating the inflammatory response, repairing the intestinal barrier by upregulating ZO‐1, Occludin and enhancing goblet cell proliferation, and microbiota regulation.

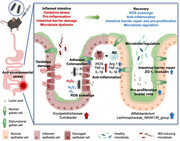

## 1 Introduction

Inflammatory bowel disease (IBD) is a type of chronic relapsing inflammatory disorder that implicates the digestive tract.^[^ ^1^, ^2^, ^3^ ^]^ Clinically, patients afflicted with IBD invariably endure recurrent manifestations such as bloody stools, abdominal distension, abdominal pain, and weight loss.^[^ ^2^, ^3^ ^]^ The severity of clinical manifestations can range from mild to severe, necessitating surgical resection of intestinal segments in severe cases, and may be accompanied by serious extraintestinal complications and even disability, seriously endangering human quality of life.^[^ ^2^, ^3^, ^4^, ^5^, ^6^, ^7^ ^]^ Presently, the treatments for IBD primarily consist of steroids, immunosuppressants, and biological agents. Nevertheless, the overall remission rate is still unsatisfactory, and there is a high recurrence rate.^[^ ^8^, ^9^, ^10^, ^11^ ^]^ Since the current treatment method is mainly accompanied by immunosuppression, some serious side effects such as severe infections and lymphoma may occur.^[^ ^12^ ^]^ Hence, it is of utmost importance to discover non‐immunosuppressive approaches to attenuate inflammation, promote mucosal healing, and reconstruct the homeostasis of the intestinal microenvironment.

During recent years, numerous studies have focused on probiotics therapy to reduce inflammation, regulate the immune system, and restore the normal intestinal microecological environment,^[^ ^13^, ^14^, ^15^ ^]^ and some probiotic strains have been employed for clinical supplement treatment of IBD.^[^ ^16^, ^17^ ^]^ However, the therapeutic effect of probiotics remains controversial due to many factors.^[^ ^18^, ^19^ ^]^ The harsh physicochemical environment of the gastrointestinal tract (e.g., gastric acids) would destroy the activity of probiotics, leading to the survival of less than the minimum effective amounts of probiotics. In addition, oxidative stress in the inflamed gut not only affects the viability of the probiotics treated by oral administration but also disrupts other probiotic flora that are already present in the gut.^[^ ^20^, ^21^, ^22^ ^]^ The dysbiosis activates the immune system, exacerbates inflammation, and generates more amount of reactive oxygen species (ROS).^[^ ^22^ ^]^ Furthermore, excessive ROS impacts the sustenance of intestinal stem cells (ISC) and the defense of intestinal epithelial cells (IEC) against harmful bacteria, further affecting intestinal flora homeostasis.^[^ ^23^, ^24^ ^]^ These detrimental factors interact with each other, forming a vicious cycle that causes continuous inflammation progression and aggravates mucosal injury. In addition, the inflamed intestine is not conducive for probiotics colonization due to factors such as a thinned mucus layer and accelerated peristalsis.^[^ ^25^, ^26^, ^27^ ^]^ In order to combat the damage to probiotics caused by the oxidative stress in IBD, the surface modification of probiotics with nano‐enzymes has emerged.^[^ ^28^ ^]^ However, few studies have addressed the protection of probiotics against the harsh set of inflammatory gastrointestinal environmental stresses described above.

Carbon dots (C dots), a carbon‐based nanomaterial with a diameter less than 10 nm and inherent fluorescence, have captured extensive attention in the biomedical field owing to their simple synthesis, good biocompatibility, low toxicity, and, more importantly, antioxidant activity.^[^ ^29^, ^30^ ^]^ The C dots have been found to play a role in scavenging ROS, alleviating inflammation, and regulating the intestinal microbiota in mouse colitis.^[^ ^31^, ^32^, ^33^, ^34^ ^]^ In addition, C dots with abundant carboxyl groups on the surface and negative charges can target and accumulate at the ulcerative sites of colitis with positive charges through electrostatic interaction.^[^ ^35^ ^]^ Moreover, carbon‐based nanomaterials can be fermented and metabolized by the intestinal microbiota into butyrate,^[^ ^36^ ^]^ which not only serves as an energy source for IECs but also upregulates the proportion of butyrate‐producing bacteria, maintains the intestinal barrier function, and regulates immune responses via Treg cells.^[^ ^37^, ^38^, ^39^ ^]^ Additionally, lactic acid, as another metabolite of intestinal flora, also promotes the proliferation and differentiation of ISCs, expediting the repair of the intestinal barrier.^[^ ^40^, ^41^ ^]^ Lactobacillus rhamnosus GG (LGG), one of the lactic acid‐producing probiotics, can restore the acidic environment of the intestine, which is conducive for maintaining a healthy ratio of beneficial bacteria to harmful bacteria.^[^ ^42^ ^]^ Moreover, P40 protein produced by LGG reduces epithelial cell apoptosis and maintains barrier function by regulating the EGFR pathway.^[^ ^43^ ^]^ The extracellular vesicles of LGG can also regulate the intestinal microbiota and alleviate mouse colitis.^[^ ^44^ ^]^

**Scheme 1:**
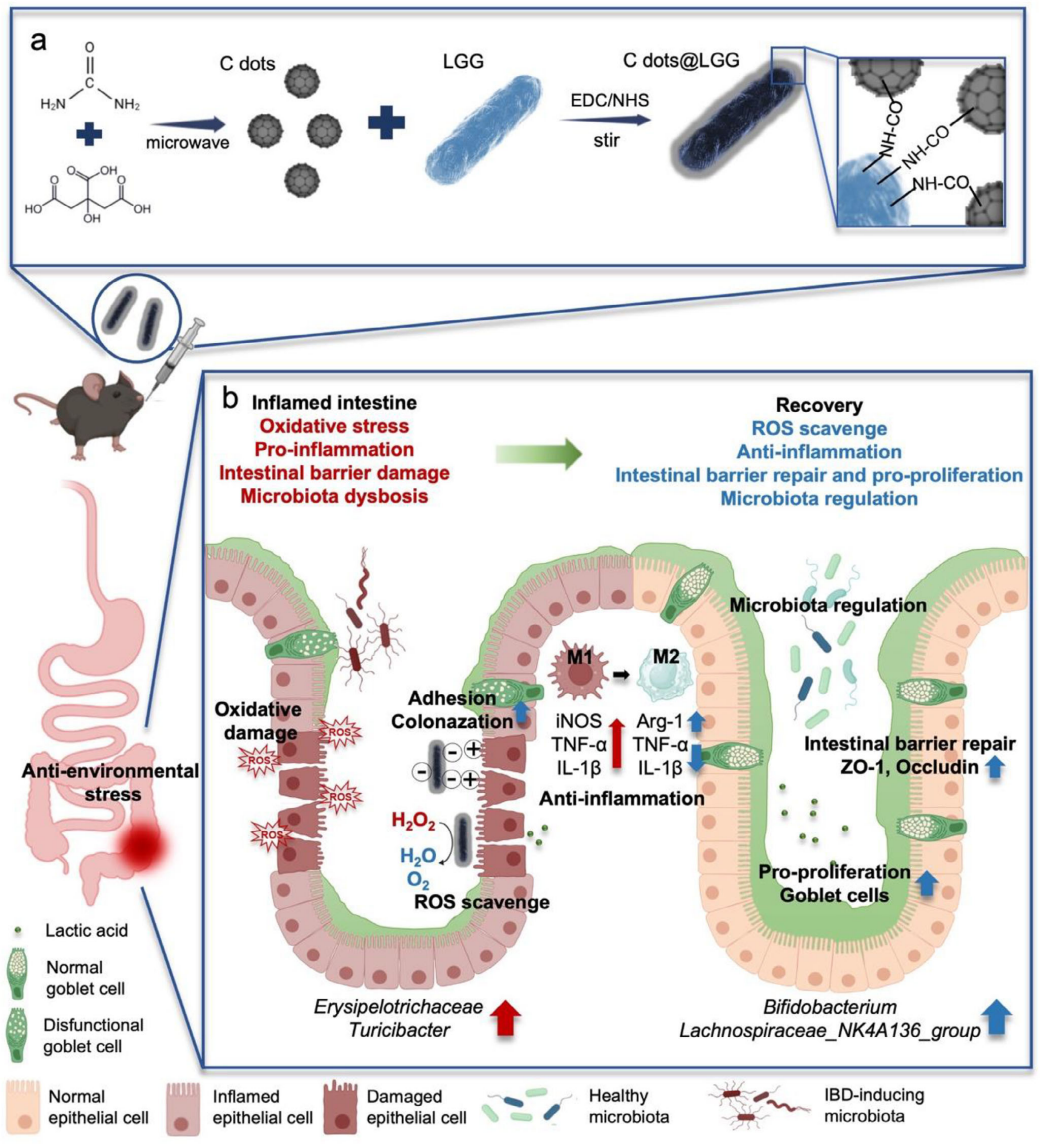
a) Schematic representation of the fabrication of carbon dots and C dots@LGG. b) C dots@LGG alleviates DSS‐induced colitis by ROS scavenging, anti‐inflammation, rebuilding the intestinal barrier and regulating the gut microbiota.

Inspired by the foregoing, we postulated that C dots possessing potent antioxidant effects could be coated on the surface of LGG to protect them from environmental hostile stressors and facilitate the probiotics in targeting and accumulating in the inflammatory site, followed by ROS scavenging and modulating a healthier metabolism status to mitigate inflammation and promote intestinal injure repair without extensive immunosuppression. Herein, chemical modification was used to encapsulate LGG with C dots coating to resist the challenge of a high ROS inflammatory environment and gastrointestinal stress, thereby increasing the survival rate of the probiotics (**Scheme** **1**a). Simultaneously, the C dots coating enhanced the adhesion and targeted colonization capability of LGG and allowed the probiotics to exert superior anti‐inflammatory ability against DSS‐induced colitis, further promoted barrier repair, and restored a healthier microecological environment (Scheme 1b). Thus, the designed C dots armed probiotics are expected to become a promising paradigm for combined non‐immunosuppressive treatment of IBD in future clinical translation.

## 2 Result and Discussion

### 2.1 Preparation and Characterization of C dots@LGG

**Figure 1:**
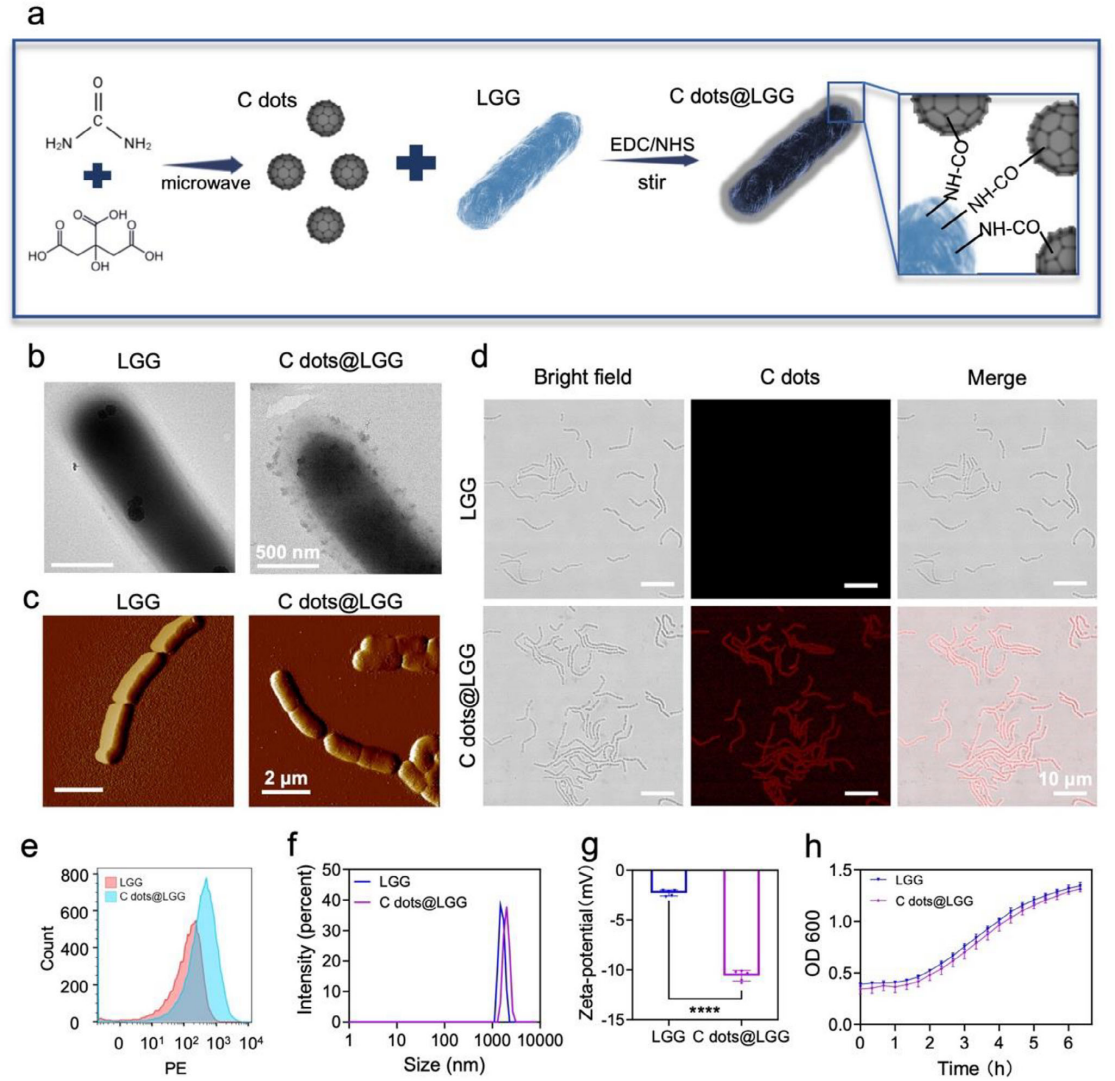
Characterization of C dots@LGG. a) Schematic representation of the fabrication process of C dots@LGG. b) TEM images of LGG and C dots@LGG. Scale bar: 500 nm. c) AFM images of LGG and C dots@LGG. Scale bar: 2 µm. d) CLSM images of LGG and C dots@LGG (C dots exhibit fluorescence under CLSM). Scale bar: 10 µm. e) Flow cytometry plot of LGG and C dots@LGG. f) Sizes of LGG and C dots@LGG. (*n* = 3) g) Zeta potentials of LGG and C dots@LGG. (*n* = 3) h) Growth curves of LGG and C dots@LGG in MRS broth. Values were mean ± SD. Statistical analysis was carried out by means of an unpaired Student's *t* test. ^***^ *p* < 0.001.

The main probiotic surface modification methods were covalently attached nanoparticles, followed by self‐polymerizing coatings and electrostatic adsorption.^[^ ^45^ ^]^ For C dots with an abundance of reactive groups on the surface, it is possible to utilize biocompatible chemical modifications to encapsulate bacteria. The carboxyl groups on C dots reacted with the amino groups on the surface of LGG to form amide bonds, thus binding the C dots onto the surface of LGG (**Figure** **1**a). The resulting C dots@LGG were observed under transmission electron microscopy (TEM) and atomic force microscopy (AFM). Both TEM and AFM images presented a coarser appearance attaching with the C‐dot nanoparticles compared with bare LGG (Figure 1b,c; Figure S1, Supporting Information). The confocal laser scanning microscopy (CLSM) images revealed that C dots exhibited red fluorescence under 560 nm laser excitation and were evenly dispersed over the surface of LGG (Figure 1d). Flow cytometry and particle size analysis of bare LGG and C dots@LGG provided further evidence that C dots had been attached to the surface of the probiotics (Figure 1e,f; Figure S2a, Supporting Information). The size of LGG was 1577 ± 209.5 nm and changed to 1975 ± 283.7 nm after being coated by C dots, whose size was 3.06 ± 0.51 nm. The C dots possessed negative charges with the ζ‐potential of ‐16.69 ± 4.05 mV, which might result from the large number of carboxyl groups present on their surface. (Figure S2b, Supporting Information). As presented in Figure 1g and C dots coating gave the probiotics more negative charges, and the ζ‐potential of LGG was reduced from ‐2.27 ± 0.31 to ‐10.58 ± 0.54 mV, which was closer to that of the C dots, enabling the probiotics to combine with the positive charges at the inflammatory site, thus promoting the targeted adhesion of LGG.^[^ ^35^ ^]^ The covalent linkage enabled C dots coating to remain on the bacterial surface for an adequate period during probiotic entry into the intestinal tract (Figure S3a, Supporting Information). According to the results, 1 × 10^9^ colony‐forming units (CFUs) of LGG were decorated with ≈47.19 µg of C dots (encapsulation efficiency of 14.88% ± 2.69%). Furthermore, plate standard counting and growth curves of bare LGG and C dots@LGG both indicated that the probiotics still possessed excellent growth activity after surface modification (Figure 1h; Figure S3b,c, Supporting Information).

### 2.2 Antioxidant, Anti‐Inflammatory, and Pro‐Proliferation Capacity of C dots@LGG

**Figure 2:**
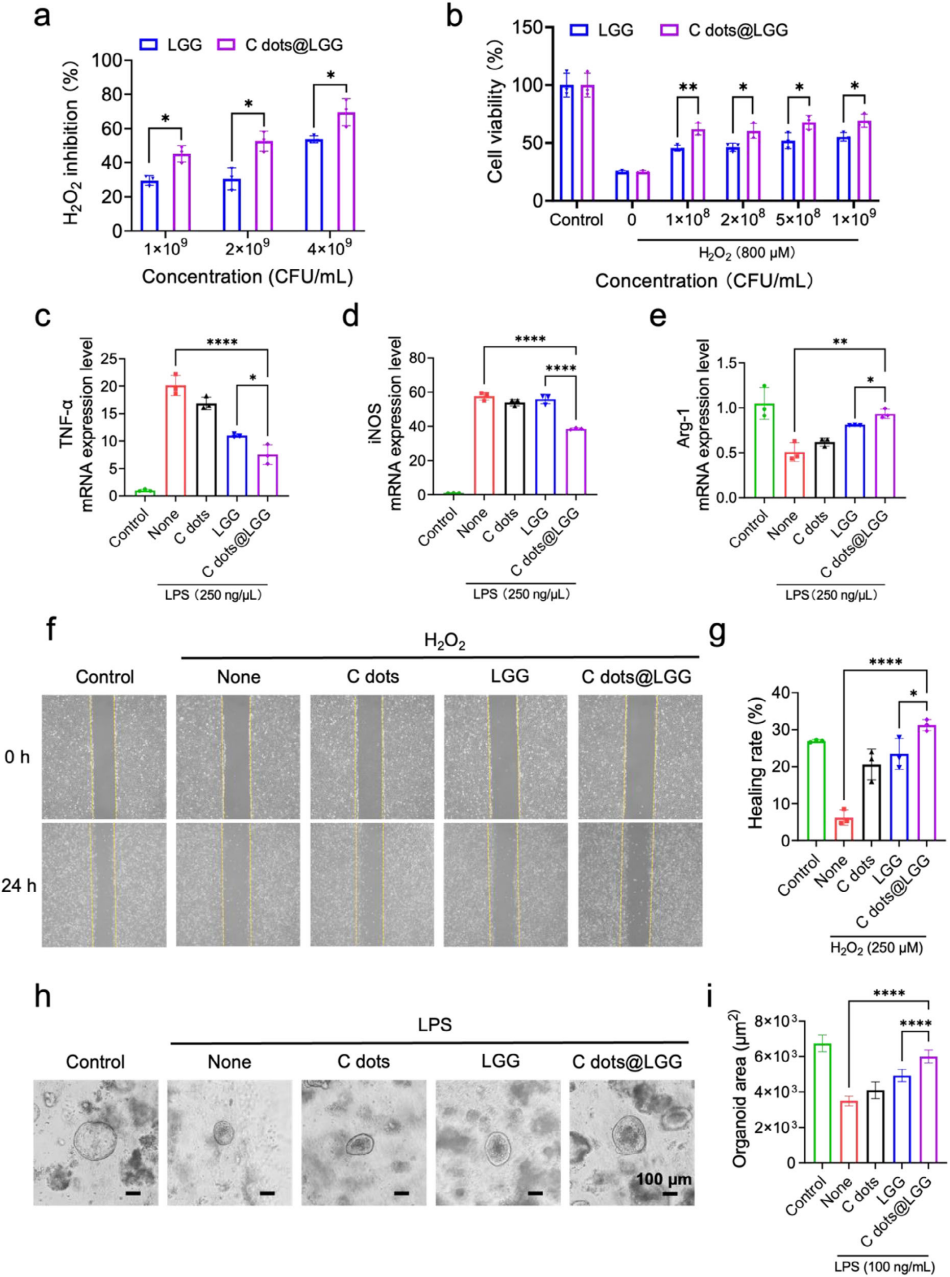
The C dots@LGG exhibited antioxidant, anti‐inflammatory, and pro‐proliferative effects in vitro. a) H_2_O_2_‐scavenging ability of LGG and C dots@LGG with different concentrations after incubated with 100 µM H_2_O_2_. b) Relative viability of caco‐2 cells after treatments with LGG and C dots@LGG with different concentrations in the presence of 800 µM H_2_O_2_. The c) TNF‐α, d) iNOS, and e) Arg‐1 levels of RAW 264.7 cells after being treated with DMEM medium, C dots, LGG, or C dots@LGG under LPS (250 ng mL^−1^) stimulation. f) Wound healing images and g) healing rates of caco‐2 cells under 250 µM H_2_O_2_ after being treated by C dots, LGG, and C dots@LGG for 24 h. h) The bright field photographs of mouse colonic organoids after being treated by C dots, LGG, or C dots@LGG for 5 days under LPS (100 ng mL^−1^) stimulation. i) Surface area statistics of colonic organoids after treatment of C dots, LGG or C dots@LGG in the presence of 100 ng mL^−1^ LPS on day 5. Values were mean ± SD (*n* = 3 for a‐e and g, *n* = 50 for i). Statistical analysis was carried out by means of one‐way analysis of variance analysis (ANOVA) and unpaired Student's *t* test. ^*^ *p* < 0.05, ^**^ *p* < 0.01 and ^****^ *p* < 0.0001.

Due to the unique properties of the C dots' surface, they have been reported to exhibit excellent antioxidant capacity.^[^ ^46^, ^47^ ^]^ The antioxidant capacity of C dots was confirmed by ABTS reagent assay, which could be enhanced with increasing concentration of C dots (Figure S4a, Supporting Information). Then, nitroblue tetrazolium (NBT) method was used to evaluate the superoxide dismutase (SOD) enzyme activity of C dots. As shown in Figure S4b (Supporting Information), the SOD inhibition rate of C dots with concentration of 80 µg mL^−1^ reached about 90%. This SOD‐like enzyme activity may be attributed to the fact that the superoxide anion is anchored to the reactive groups of C dots (Figure S2c, Supporting Information) such as carboxyl, hydroxyl, and amine groups by hydrogen bonding and provides electrons to form a p‐π conjugate with the large π‐system (C═C/C═N), which is then followed by the extraction of electrons from the other superoxide to produce H₂O₂ to restore the original C‐dot structure.^[^ ^46^, ^48^ ^]^ We also assessed the ability of scavenging H_2_O_2_ of C dots. After incubating with 100 µm H_2_O_2_, the C dots with 200 µg mL^−1^ could eliminate 88.2% of H_2_O_2_ (Figure S4c, Supporting Information). To determine whether C dots endowed LGG with stronger ROS scavenging ability, LGG and C dots@LGG of different concentrations (with equal bacterial count) were co‐incubated with 100 µM H_2_O_2_. The content of remaining H_2_O_2_ showed that LGG modified by C dots had a stronger efficiency in scavenging H_2_O_2_ than bare LGG (**Figure** **2**a). Then, the in vitro biocompatibility of C dots and C dots@LGG was assessed in caco‐2 and RAW 264.7 cells. After treated by C dots and C dots@LGG with different concentrations, both two cell lines remained more than 70% cell viability and the difference of cell viability between C dots@LGG and bare LGG was not obvious, indicating that the introduction of C dots did not increase cytotoxicity (Figures S5 and S6, Supporting Information), which ensured the potential biomedical application of C dots@LGG.

Subsequently, the protection ability of C dots and C dots@LGG against oxidative stress was evaluated by constructing an oxidative stress environment using H_2_O_2_‐treated caco‐2 cells. The survival rate of the cells dropped sharply to 25.1% after being cultured in 800 µM H_2_O_2_ for 4 h, while C dots@LGG could rescue it to 62.1%–69.3% with different concentrations, which was higher than that of LGG or C dots (Figure 2b; Figure S7, Supporting Information). The calcein/propidium iodide (Calcein/PI) staining images of 600 µM H_2_O_2_‐stimulated caco‐2 cells after different treatments for 4 h also proved that C dots@LGG protected the cells from oxidative damage (Figure S8, Supporting Information), which was also confirmed by the scavenging of intracellular ROS (Figure S9, Supporting Information).

Furthermore, the anti‐inflammatory ability of C dots@LGG was also assessed. C dots (250 ng mL^−1^), C dots@LGG (1 × 10^8^ CFU mL^−1^), or LGG (equal bacterial count with C dots@LGG) were cultured with lipopolysaccharides (LPS) stimulated RAW 264.7 cells for 12 h. As shown in Figure 2c–e, the C dots@LGG markedly reduced the expression of TNF‐α and iNOS (i.e., proinflammatory cytokines and a biomarker of M1 macrophage) and upregulated the anti‐inflammatory biomarker Arg‐1(i.e., a biomarker of M2 macrophage). Thus, the designed C dots@LGG induces a decrease in ROS‐induced inflammatory injury and conversion of M1 cells to M2, and its anti‐inflammatory capacity is superior to that of C dots and bare LGG.

Alternatively, the antioxidant effects of C dots and the biological effects of LGG were expected to have a reparative effect on pre‐existing damage. The pro‐proliferation capability at the cellular level of C dots@LGG was evaluated by the wound healing assay. C dots (250 µg mL^−1^), bare LGG, or C dots@LGG (with equal bacterial count of 1 × 10 ^8^ CFU mL^−1^) were treated with scratched caco‐2 cells under 250 µm H_2_O_2_ for 24 h. The representative images of the scratches (Figure 2f) and healing rates (Figure 2g) showed that H_2_O_2_ inhibited the migration of caco‐2 cells with only 6.2% of scratched area recovering, while the C dots@LGG group exhibited significant migration promotion and the healing rate increased to 31.2%, better than the 23.4% for bare LGG group and 20.6% for C dots group. To further confirm the pro‐proliferation effect of C dots@LGG, mouse colonic organoids were constructed and stimulated by 100 ng mL^−1^ LPS to mimic an inflamed intestinal condition in vitro. Then they were treated with C dots (250 µg mL^−1^), bare LGG or C dots@LGG (with equal bacterial count of 1 × 10^8^ CFU mL^−1^) for 5 days. The representative images presented that C dots@LGG promoted the growth condition of colonic organoids with a larger surface area than C dots and bare LGG groups (Figure 2h). The statistic of organoid area further confirmed the results by promoting the surface area of organoids to increase from 3497.5 µm^2^ in the LPS group to 5998.9 µm^2^ in the C dots@LGG group, superior to 4104.3 µm^2^ in the C dots group and 4931.6 µm^2^ in the bare LGG group. The antioxidant, anti‐inflammatory, and pro‐proliferation capacity of C dots@LGG shown above suggested that the modification of C dots to LGG not only reduced the oxidant damage to intestinal cells but also provided a milder environment for LGG to better exert its functions to reduce the inflammation and boost the proliferation.

### 2.3 Enhanced Resilience Against Gastrointestinal Stresses and Augmented Retention Proficiency of C dots@LGG

**Figure 3:**
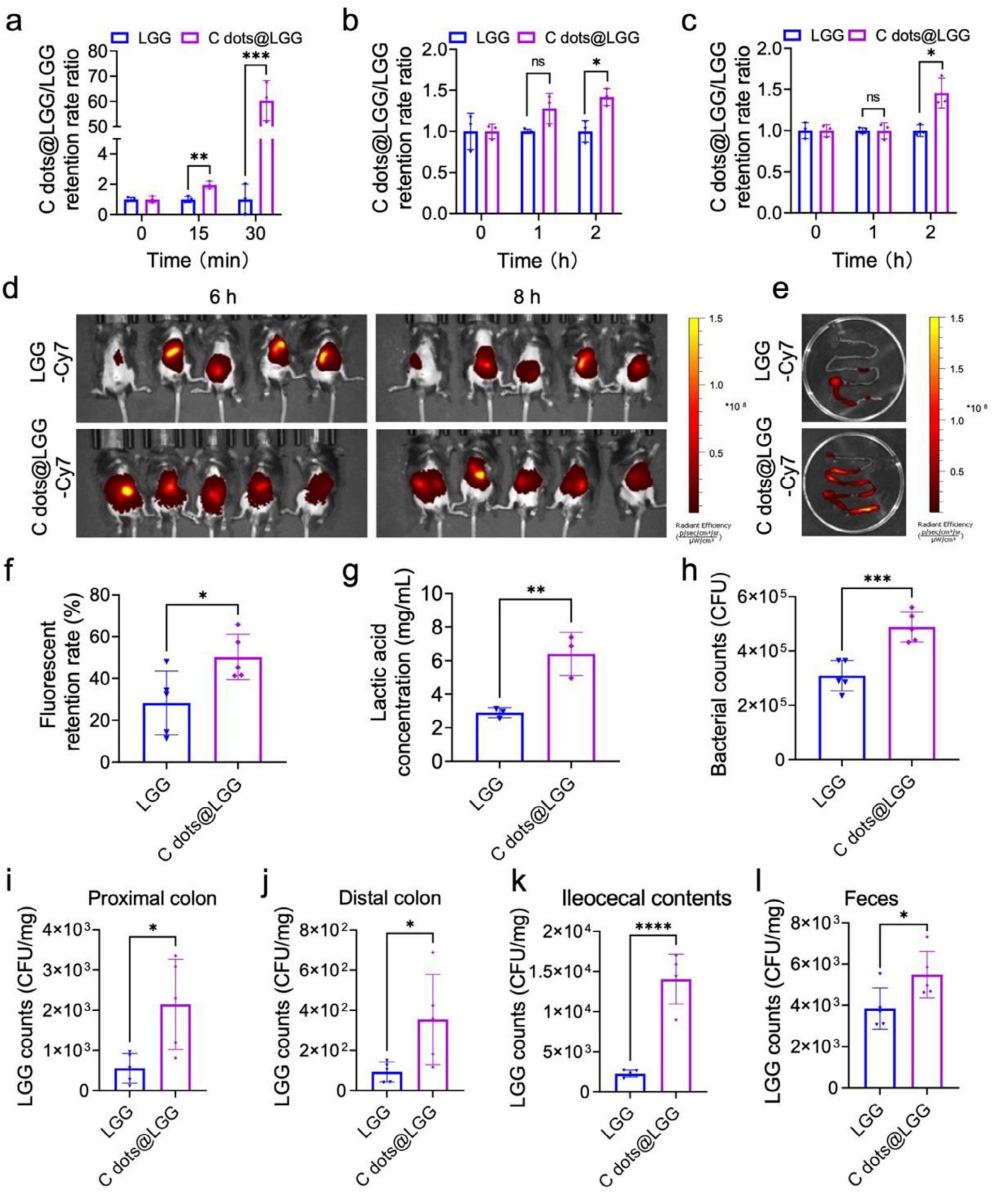
C dots@LGG exhibited the capability of gastrointestinal environmental stress resistance and enhanced intestinal colonization. Survival rate of LGG and C dots@LGG after exposure to a) SGF (pH 2.5), b) SIF, and c) SICF (with 200 µM H_2_O_2_). d) In vivo fluorescence images were presented to evaluate the retention of LGG and C dots@LGG in colitis mice gavaged by 2 × 10^8^ CFU of Cy7‐labeled LGG and C dots@LGG. e) Ex vivo fluorescence images of the intestines of mice with colitis treated by Cy7‐labeled LGG and C dots@LGG after 8 h. f) Retention rate of LGG and C dots@LGG at 8 h in colitis mice after gavage. The average fluorescence intensity at 4 h was taken as the reference base. g) The amount of lactic acid in the supernatants of different groups co‐incubated with intestinal flora extracted from healthy wild C57BL/6 mouse for 3 days in the presence of 200 µM H_2_O_2_. h) Bacterial counts of LGG and C dots@LGG adhering to caco‐2 cells after co‐incubation for 3 h. The number of LGG in i) proximal colon, j) distal colon, k) ileocecal faeces, and l) faeces in colitis mice at 24 h after being gavaged by 2 × 10^8^ CFU LGG or C dots@LGG. Values were mean ± SD (*n* = 3 for a‐c and g; *n* = 5 for f, h‐l). Statistical analysis was carried out by means of one‐way ANOVA and unpaired Student's *t* test. ^*^ *p* < 0.05, ^**^ *p* < 0.01, ^***^ *p* < 0.001 and ^****^ *p* < 0.0001, ns stands for not significant.

To fulfill the excellent antioxidant, anti‐inflammatory, and pro‐proliferation capacity of C dots@LGG in vivo, it is essential to develop highly viable probiotics after transmitting through the gastrointestinal tract and deliver probiotics to the diseased site with longer retention time in the intestine.^[^ ^49^ ^]^ The resistance ability against the gastrointestinal assaults of C dots@LGG was first evaluated by measuring the survival rate of LGG in simulated digestive solution using the plate counting method. As is widely acknowledged, the pH level of the gastric fluids exhibits acidity ranging from pH 1.5 to 3.5, which ascends to around pH 7–8 in the distal jejunum and ileum, before transitioning to pH 5–8 in the colon, accompanied by a significant degree of interindividual variability.^[^ ^50^, ^51^ ^]^ As the liquid generally passes through the stomach within 20–30 min and within ≈2 h in both small and large intestine, respectively,^[^ ^28^, ^51^ ^]^ the same amount of LGG and C dots@LGG (1 × 10^9^ CFU mL^−1^) were incubated in simulated gastric fluid (SGF) for 30 min, in simulated intestinal fluid (SIF), and simulated inflammatory colon fluid (SICF) containing 200 µM H_2_O_2_ for 2 h, respectively.^[^ ^28^ ^]^ The survival rate of C dots@LGG in SGF was 60.34 times higher compared to bare LGG (**Figure** **3**a; Figure S10, Supporting Information). According to a previous study, LGG was viable after incubation in SGF at pH 3.0–7.0, but not at pH less than 3.0.^[^ ^52^ ^]^ In contrast, it was found that C dots@LGG had a good chance to survive in SGF with a pH below 3.0, according to our results. In the meantime, the survival rate of C dots@LGG was 1.42 times in SIF (Figure 3b; Figure S11, Supporting Information) and 1.46 times (Figure 3c) in SICF in comparison with bare LGG. The C dots coating may provide a partial physical barrier for probiotics. The abundant functional groups on the surface of C dots may also exert acid‐base buffering effects. For example, H^+^ could be captured by ‐COO^‐^ and ‐NH₂ groups, thereby regulating the pH of the local microenvironment and reducing the erosion of probiotics by gastrointestinal fluids.^[^ ^26^ ^]^ Additionally, the antioxidant properties conferred by the coating mitigated the damage to probiotics caused by the highly oxidative inflammatory environment, thereby enhancing their survival rate.

Further, we explored whether C dots@LGG could maintain viability in an inflammatory microecological environment. The intestinal microbiota of healthy mice was collected and cultured in medium containing 200 µM H_2_O_2_ to mimic the inflammatory microecological environment. Subsequently, 2 × 10^8^ CFU of LGG or C dots@LGG were added and co‐cultured for three days. As the major product of LGG, the lactic acid in the supernatant was then detected by HPLC. As shown in Figure 3g and C dots@LGG group produced more lactic acid than the LGG group, indicating that C dots@LGG survived more LGG and kept a better metabolic viability under inflammatory microecological circumstances. Subsequently, we co‐cultured caco‐2 cells and LGG or C dots@LGG (both 1 × 10^7^ CFU) for 3 h to examine the adhesion ability of C dots@LGG. After removing the floating probiotics and lysing caco‐2 cells, the number of probiotics was validated by the plate counting method, and the result suggested the number of adhered C dots@LGG was 1.58 times higher than that of LGG (Figure 3h). All these results suggested that C dots@LGG had the potential to transit through the gastrointestinal tract with better viability and adhesion ability.

Then, in vivo retention and colonization capacity of C dots@LGG in the DSS‐induced colitis mice intestine were investigated. C57BL/6 mice were given free drinking water with 4% DSS for 7 days for the induction of colitis, which then were orally administered with Cy7‐labeled LGG and C dots@LGG (with the same amount of 2 × 10^8^ CFU) on day 7. The fluorescence intensity of probiotics at a series of time points was collected. As indicated in Figure 3d–f, the fluorescent retention rate (the fluorescence intensity at 4 h was taken as the baseline) in the inflamed mice and intestine tissue of C dots@LGG was markedly exceeded that of the LGG group at 8 h after oral administration, suggesting the superior intestinal retention of C dots@LGG. Meanwhile, equal LGG and C dots@LGG content of 1 × 10^9^ CFU mL^−1^ was given by gavage to the colitis mice, and the number of probiotics in their proximal colon tissues, distal colon tissues, cecal contents, and feces was determined by the plate counting method. As displayed in Figure 3i–l, the probiotic number in the proximal colon tissues, distal colon tissues, cecal contents and feces of C dots@LGG group was 3.84, 8.60, 6.18 and 1.43 times as many as that of LGG group at 24 h after gavage, respectively, suggesting that C dots@LGG with negative charges on the surface could facilitate the probiotics to be colonized and retained on the inflamed colon tissue with cationic charges.^[^ ^49^ ^]^ Moreover, the hydroxyl and carboxyl groups on the surface of C dots may form hydrogen bonds with sugar chains and mucins on intestinal cell surfaces, thereby enhancing adhesion.^[^ ^53^ ^]^ In addition, there were more probiotics in cecal contents and proximal colon tissues than in other parts of the colon tissue, which may be attributed to the unique functional regions of the colon. Namely, the cecum and proximal region of the colon are the main sites of fermentation, while the distal colon is mainly for extracting liquids and electrolytes.^[^ ^54^ ^]^ The accumulation of probiotics in these parts can provide a better environmental condition for probiotics to regulate microecological metabolism. In general, the developed C dots‐modified probiotics manifested an enhanced survival rate and viability within the austere environment of the gastrointestinal tract and possessed stronger adhesion capacity, thereby resulting in efficient retention and colocalization in the inflamed colon.

### 2.4 Synergistic Amelioration Efficacy of C dots@LGG on DSS‐Triggered Mouse Colitis

**Figure 4:**
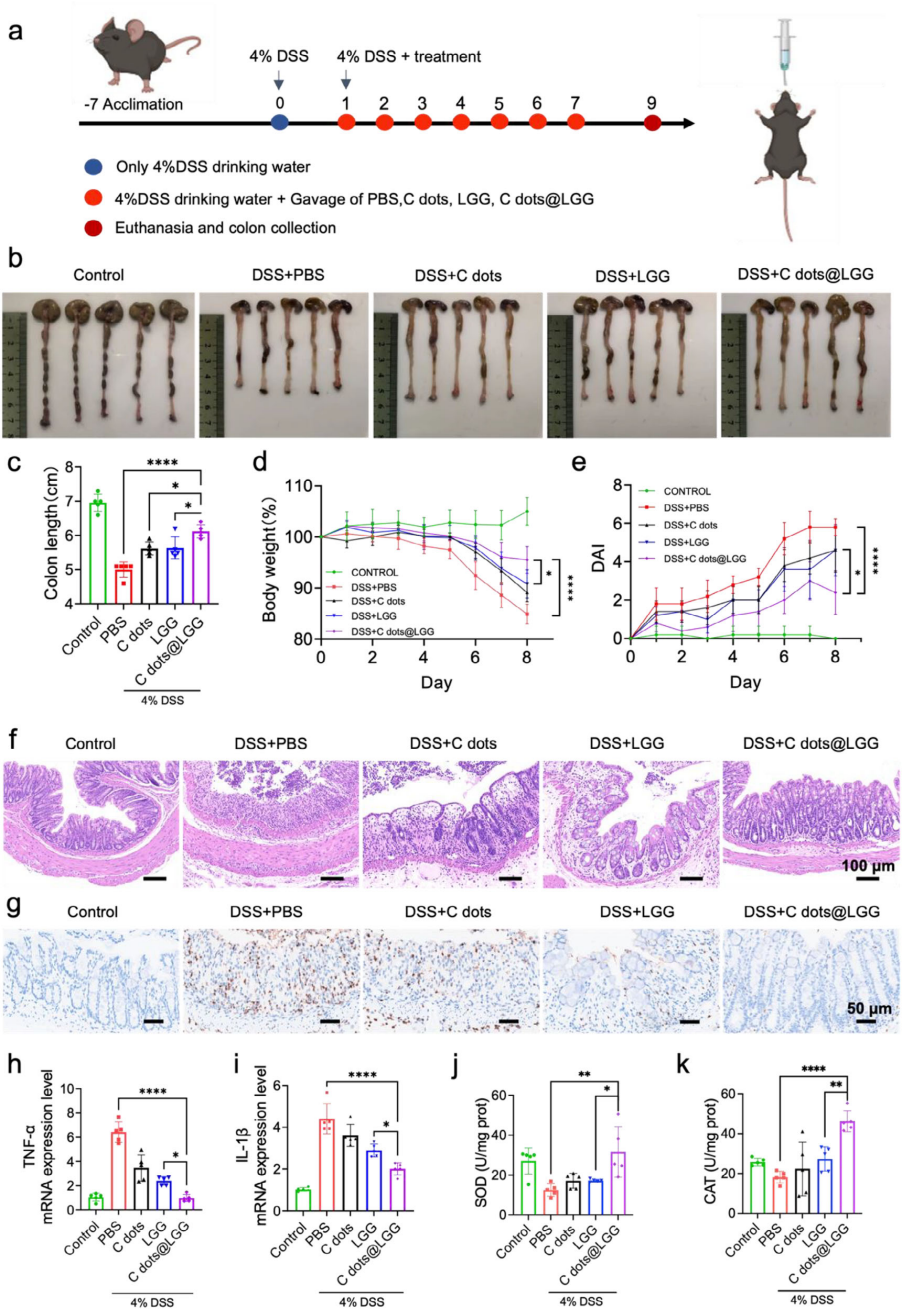
Synergistic amelioration efficacy of C dots@LGG on DSS‐triggered mouse colitis. a) Illustration of experimental protocol. b) Typical pictures and c) colon lengths data of colons obtained from mice treated by PBS, C dots, LGG, or C dots@LGG on day 9. d) Body weight curves, e) disease active index (DAI) of mice treated by PBS, C dots, LGG, or C dots@LGG, respectively. f) Typical hematoxylin & eosin (H&E) staining images of distal colon tissues from mice in control, DSS model, C dots, LGG, and C dots@LGG group on day 9. Scale bar: 100 µm. g) Typical immunochemistry images of MPO from different groups. Scale bar: 50 µm. Relative mRNA level of TNF‐α h) and IL‐1β i) of colon tissues from mice treated by PBS, C dots, LGG, and C dots@LGG. The content of SOD j) and CAT k) in colon tissues from mice treated by PBS, C dots, LGG and C dots@LGG. Values were mean ± SD (*n* = 5 for c–e and h–k). Statistical analysis was carried out by means of one‐way ANOVA. ^*^ *p* < 0.05, ^***^ *p* < 0.001 and ^****^ *p* < 0.0001, ns stands for not significant.

In light of the aforementioned advantages of C dots@LGG, we next explored the synergistic alleviation effect in DSS‐induced colitis in vivo. As shown in **Figure** **4**a, male C57BL/6 mice were evenly partitioned into 5 groups, and 4% DSS was given in free drinking water to construct an acute colitis model. The mice in each group were given an equal volume of PBS, C dots (8 mg kg^−1^ body weight), LGG, and C dots@LGG (bacterial dose, 1 × 10^9^ CFU mL^−1^), respectively, from day 1 to day 7. Daily recordings were made for body weight as well as the disease activity index (DAI) of each mouse to reflect disease manifestation changes. As demonstrated in Figure 4d,e, the model group had significant weight loss and the highest DAI score, reflecting a successful inauguration of acute colitis model, while the mice in the other three groups with different treatment presented varying degrees of improvement. Compared with the mice in the LGG and C dots group, the C dots@LGG‐treated mice showed a decreased trend of weight loss and significantly less loss of weight loss on day 8 (Figure 4d). In the meantime, the DAI score of C dots@LGG‐treated mice was also lower than mice in other treatment groups (Figure 4e). Since colon shortening represents an obvious outward indication of colitis, both the configuration of mice colons and the statistical details of colon length demonstrated that the colon length of the C dots@LGG group exceeded that of the model group and other treatment groups. (Figure 4b,c), indicating that C dots@LGG markedly alleviated colitis in external manifestation.

According to the H&E staining images, C dots@LGG also exhibited the best therapeutic effects than other treatment groups at the histological level. As shown in Figure 4f and Figure S12 (Supporting Information), the model group manifested epithelial loss, crypt destruction, and massive infiltration of inflammatory cells, which were restored to varying degrees after being treated by C dots or LGG. Remarkably, these destructive tissue manifestations were significantly restored in the C dots@LGG group, which were almost recovered to the standard of the healthy control group. The statistical result further confirmed this superiority (Figure S13a, Supporting Information). Subsequently, the content of intestinal myeloperoxidase (MPO) was measured, which is primarily expressed by neutrophils and reflects the level of inflammation. The results in Figure 4g and Figure S13b (Supporting Information) showed that the content of MPO was significantly increased in the DSS group, but was notably mitigated following C dots@LGG administration. Meanwhile, compared with the LGG or C dots group, the C dots@LGG group exhibited a relatively lower level of the pro‐inflammatory cytokines TNF‐α (Figure 4h) and IL‐1β (Figure 4i). Both results illustrated the excellent anti‐inflammatory capacity of C dots@LGG and its superiority over bare LGG. In addition, the effect of ROS scavenging in the colon of mice was explored after C dots@LGG administration. As shown in Figure 4j,k and Figure S13c (Supporting Information), compared with the DSS and other treatment groups, the level of malondialdehyde (MDA) was significantly decreased after C dots@LGG administration, while the activities of SOD and catalase (CAT) were significantly enhanced, indicating that C dots@LGG administration could effectively alleviate oxidative stress in IBD mice. Finally, a histological examination of the principal organs revealed no notable disparities on day 8 (Figure S14, Supporting Information), compared to healthy control group mice, indicating good biosafety and fewer side effects of C dots@LGG.

### 2.5 Intestinal Barrier Repair and Promoting Proliferation Effect of C dots@LGG

**Figure 5:**
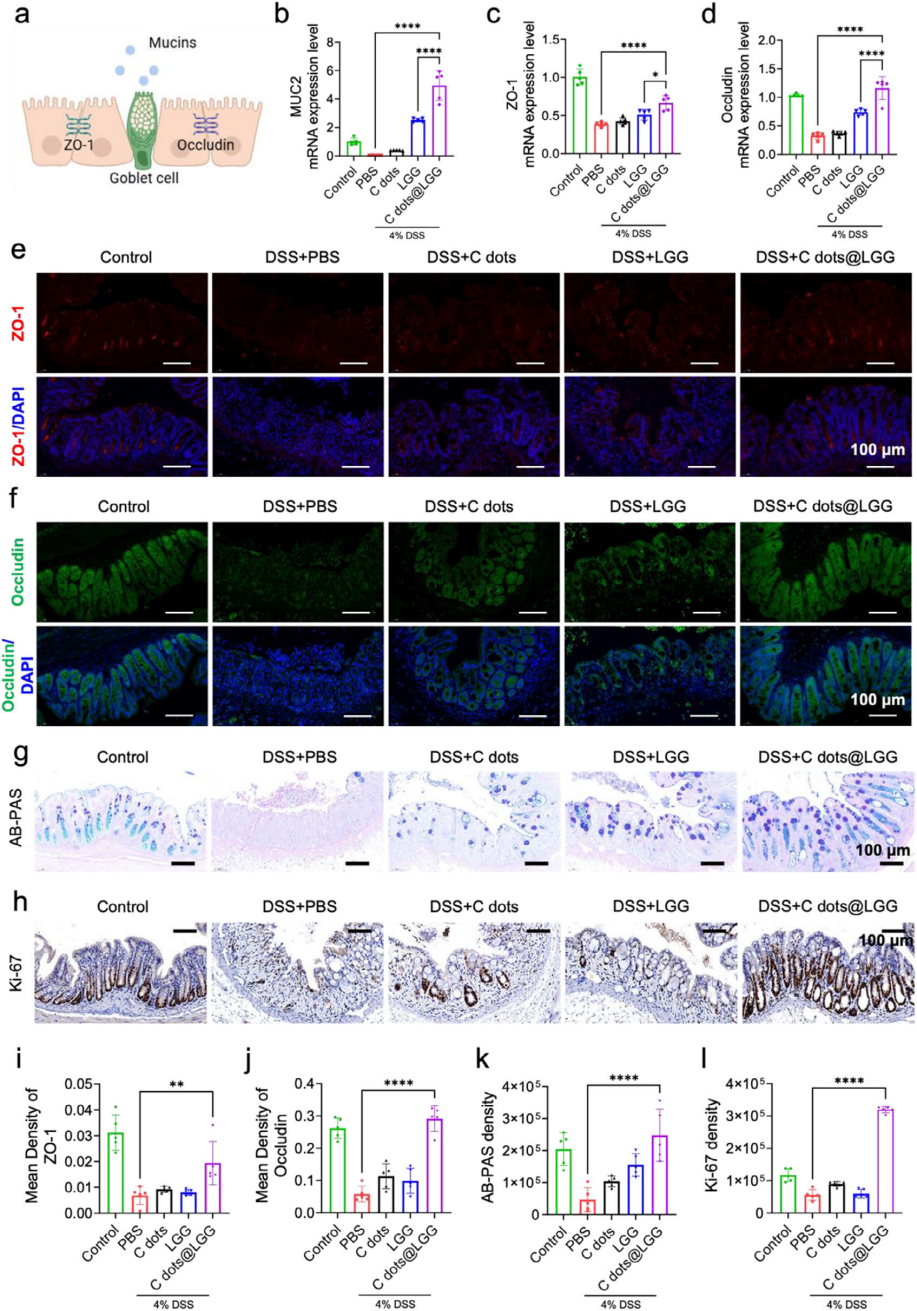
Intestinal barrier repair and pro‐proliferative capabilities of C dots@LGG in vivo. a) Illustration of the intestinal barrier. b) MUC2, c) ZO‐1, and d) Occludin mRNA expression levels were evaluated by qPCR. Exemplary IF staining images depicting the expression of TJ proteins, specifically e) ZO‐1 and f) Occludin. ZO‐1 presented as red fluorescence, and Occludin presented as green. Scale bar: 100 µm. g) Representative images of AB‐PAS staining and h) immunohistochemical staining of Ki‐67 of colon tissues from mice treated by PBS, C dots, LGG, or C dots@LGG. Scale bar: 100 µm. Mean density of i) ZO‐1, j) Occludin, k) AB‐PAS, and l) Ki‐67 in colon sections calculated by ImageJ software. Values were mean ± SD (*n* = 5). Statistical analysis was carried out by means of one‐way ANOVA. ^*^ *p* < 0.05, ^**^ *p* < 0.01 and ^****^ *p* < 0.0001.

As various substances pass through the large intestine every day, an ingenious barrier system spatially segregates harmful substances and host immune cells to avoid excessive immune responses. The mucosal barrier system in the large intestine is formed by three physical barriers, which are the mucus layer produced by goblet cells, the glycocalyx on IECs, and cell junctions, including tight and adhesion junctions, such as zonula occludens‐1 (ZO‐1) and Occludin (**Figure** **5**a).^[^ ^55^ ^]^ However, in the pathological condition of IBD, excessive oxidative stress disrupts the mucosal barrier system, and an excessive amount of harmful substances enter the lamina propria, which activates immune cells and triggers an immune response, thereby aggravating inflammation.^[^ ^20^, ^21^, ^22^ ^]^ Consequently, reconstructing and maintaining the integrity of the intestinal barrier is of utmost importance for the treatment of IBD. Thus, the condition of mucus expression was examined. MUC2 is the main component to forms the gut mucus skeleton. As demonstrated in Figure 5b, the relative mRNA expression of MUC2 in the DSS group was almost depleted, while that of the C dots@LGG group was considerably increased. Similar outcomes were manifested in the AB‐PAS staining images. As shown in Figure 5g, no visible goblet cells were found in the DSS group, while the C dots@LGG group boasted nearly intact glandular ducts and crypts. The quantity of goblet cells was significantly replenished, which was almost on a par with that of the healthy control group (Figure 5g,k).

Subsequently, quantitative polymerase chain reaction (qPCR) and immunofluorescence (IF) staining were utilized to evaluate the relative mRNA and protein expressions of ZO‐1 and Occludin (Figure 5c–f,i,j). The results revealed that the level of ZO‐1 and Occludin of the C dots@LGG group was surpassing those of the DSS model group, C dots group, and bare LGG group, illustrating the superiority of C dots@LGG in intestinal barrier repair. In addition, the expression of Ki‐67 in the colon was evaluated. The immunohistochemical staining showed an extraordinary increase of Ki‐67 in the C dots@LGG group, which was surpassing that in the DSS group and other treatment groups and even higher than that in the healthy control group (Figure 5h,l). This may be due to an active response to the IEC damages induced by DSS, indicating a high pro‐proliferation capacity of C dots@LGG to rebuild and maintain the intestinal barrier integrity. Based on the above results, the C dots@LGG exhibited a better amelioration effect on DSS‐triggered colitis by its outstanding effects on intestinal barrier repair and promoting proliferation.

### 2.6 Regulation of Gut Microbiota by C dots@LGG in DSS‐Induced Colitis Mice

**Figure 6:**
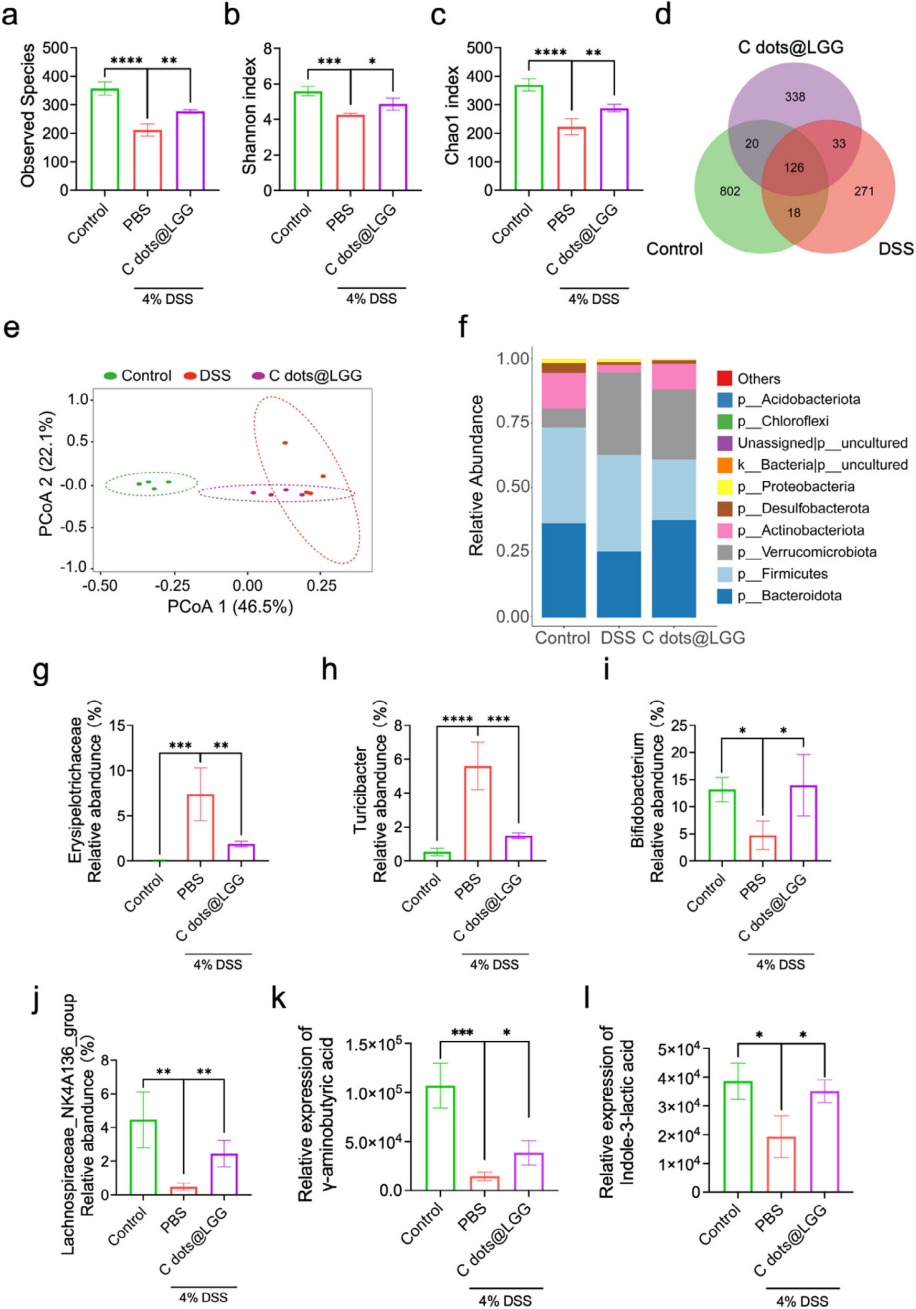
Regulation of bacterial homeostasis by C dots@LGG. Gut microbiota in colitis mice after C dots@LGG treatment was examined by 16S rDNA sequencing. The stool samples were gathered on day 8. a–c) The α‐diversity of intestinal micro‐organisms was evaluated by observed species, Shannon index, and Chao1 index. d) Venn diagram of intestinal bacterial species detected in the healthy control group, model group, and C dots@LGG group. e) β‐diversity of the microbial community in the healthy control group, model group, and C dots@LGG group through PCoA. f) The top ten dominant phylum of the intestinal microbiota of the healthy control group, DSS model group, and C dots@LGG group. Relative abundance of g) *Erysipelotrichaceae*, h) *Turicibacter*, i) *Bifidobacterium*, and j) *Lachnospiraceae_NK4A136_group*. The relative expression of γ‐aminobutyric acid k) and indole‐3‐lactic acid l). Values were mean ± SD (*n* = 4). Statistical analysis was carried out by means of one‐way ANOVA and unpaired Student's t test. ^*^ *p* < 0.05, ^**^ *p* < 0.01, ^***^ *p* < 0.001 and ^****^ *p* < 0.0001.

It is well known that there is abundant commensal microbiota existing in the intestine. Under healthy circumstances, the interplay between the intestinal microbiota and the host can attain a subtle equilibrium. The intestinal microbiota assists in digesting nutrients and generates metabolites that can mediate the activity of the immune system and IECs. Concurrently, an undamaged intestinal barrier precludes direct interaction between intestinal microorganisms and IECs as well as immune cells, thereby avoiding aberrant immune responses. When the balance is disrupted in colitis condition, increased pathogens will pass through the damaged intestinal barrier and directly activate immune cells, triggering various abnormal immune responses. On the contrary, the beneficial bacteria may help rebuild the barrier and restore balance to alleviate inflammation.^[^ ^55^ ^]^ Thus, the fecal micro‐organisms of mice in healthy control, DSS model, and C dots@LGG group were analyzed to investigate the effect of C dots@LGG on the regulation of gut microbiota in colitis conditions. The analysis of observed species in fecal samples revealed that the administration of DSS significantly reduced observed species richness, which was notably increased after the treatment of C dots@LGG (**Figure** **6**a). As expected, the α‐diversity of gut microbiota in the C dots@LGG group (Shannon and Chao1 index) was also increased (Figure 6b,c). The Venn diagram also exhibited that the administration of C dots@LGG enlarged the number of species of gut microbiota compared to the DSS model group (Figure 6d). β‐diversity is used to study the difference in community composition. To investigate the variances and overall similarity of gut microbiota, the principal coordinates analysis (PCoA) diagram based on Bray‐Curtis distance mictrix was utilized. Similar to the outcomes aforementioned, the PCoA diagram demonstrated that the colitis mice treated with C dots@LGG tended to be closer to the healthy mice in comparison with mice from the model group (Figure 6e). Although there was partial overlap between the model group and the C dots@LGG group, which might be attributed to the relatively short intervention duration, it still indicated that oral administration of C dots@LGG could positively restore the healthy intestinal microecological composition in colitis mice.

Next, the top 10 dominant phylum of the intestinal microbiota were displayed in Figure 6f. The relative abundance of *Bacteroidota* decreased in the DSS model group, while it recovered to a level approximating that of the healthy control group after oral administration with C dots@LGG. For a profound study, we further examined the composition of various colonic microorganisms in healthy controls, model, and C dots@LGG group at the family and genus levels. As shown in Figure 6g, at the family level, oral administration of C dots@LGG significantly reduced the relative abundance of *Erysipelotrichaceae*, which might be increased in colitis mice as a potential pathogen according to previous studies.^[^ ^56^, ^57^ ^]^ Concurrently, the DSS model group showed an upregulation of *Turicibacter* and a downregulation of *Bifidobacterium* and *Lachnospiraceae_NK4A136_group* at the genus level (Figure 6h–j). *Turicibacter* is a conditioned pathogen that might increase in both colitis mice and inflammation‐induced colorectal cancer mice.^[^ ^58^, ^59^ ^]^ *Bifidobacterium* and *Lachnospiraceae_NK4A136_group* are well‐known probiotics that *Bifidobacterium* can produce lactic acid to keep an acidic environment to limit the viability of pathogens and compete with harmful bacteria for nutrients and adhesion sites,^[^ ^60^ ^]^ and *Lachnospiraceae_NK4A136_group* can produce butyrate and maintain the balance of intestinal microecology.^[^ ^61^, ^62^ ^]^ In contrast, the C dots@LGG oral administration considerably diminished the richness of *Turicibacter* but upregulated *Bifidobacterium* and *Lachnospiraceae_NK4A136_group*. Then, we used untargeted metabolomics to analyze gut microbiome metabolites and found that the relative expression levels of γ‐aminobutyric acid (GABA) and indole‐3‐lactic acid (ILA) in the C dots@LGG group were higher than those in the DSS model group (Figure 6k,l). As metabolites of Bifidobacterium, these two compounds were consistent with the changes in the intestinal microbiota. As an important neurotransmitter, GABA can inhibit the production of IL‐17A to alleviate colitis^[^ ^63^ ^]^ while ILA can reduce the aggregation of inflammatory macrophages by inhibiting the production of CCL2/7 in epithelial cells, thereby alleviating colitis.^[^ ^64^ ^]^ Overall, it was found that the C dots@LGG could adjust the gut microbiota of mice with colitis to a relatively healthy state, thereby maintaining gut microbial homeostasis and alleviating colitis.

## 3 Conclusion

To sum up, a collaborative therapy platform was developed by modifying C dots onto the surface of probiotic LGG, which could endow probiotics with potent antioxidant effects to provide protective effects against the environmental stresses and oxidative damages, enabling probiotics to target and colonize in the inflamed area. The C dots coating significantly enhanced the anti‐inflammation effect of LGG in DSS‐induced colitis. Meanwhile, the C dots@LGG, with better metabolic viability to produce lactic acid, promoted the proliferation of colonic organoids and intestinal barrier repair in colitis colon, which indicated that the C dots@LGG possessed the ability to boost mucosal healing. Furthermore, the C dots@LGG regulated the microbial community by enriching the diversity of gut microbiota and providing a mild microenvironment for beneficial bacteria growth and inhibiting that of harmful bacteria. Altogether, the C dots@LGG presented a superb alleviation effect against DSS‐induced colitis. In a word, the C dots@LGG has the potential to serve as a non‐immunosuppressive therapeutic paradigm for the synergistic alleviation of IBD through resistance to oxidative stress, promotion of mucosal healing, and modulation of the gut microbiota.

## Conflict of Interest

The authors declare no conflict of interest.

## Supporting information



Supporting Information

## Data Availability

The data that support the findings of this study are available from the corresponding author upon reasonable request.
