# Supplementary material for: Carbon‐Coated Probiotics Restore Intestinal Homeostasis to Promote Damage Repair in Inflammatory Bowel Disease
Source: Adv Sci (Weinh). 2025 Jul 12;12(37):e09812. doi: 10.1002/advs.202509812 (PMC12499399; doi:10.1002/advs.202509812)
Supplement: Supplementary file 1 — Supporting Information [file ADVS-12-e09812-s001.docx]

Supporting Information

**Carbon-coated probiotics restore intestinal homeostasis to promote damage repair in** **inflammatory bowel disease**

Xue Chen^a, #^, Haiyan Guo^b, #^ Yuhan Li^b^, Qiaowen Lin^a^, Hua Liu^a^, Liwen Hong^a^, Lei Wang^c,^ *, Jie Zhong^a,^ *, Dalong Ni^b,^ *, Zhengting Wang^a,^ *

^a^Department of Gastroenterology, Ruijin Hospital, Shanghai Jiao Tong University School of Medicine, No. 197, Ruijin 2^nd^ Rd, Shanghai, 200025, PR China

^b^Department of Orthopaedics, Shanghai Key Laboratory for Prevention and Treatment of Bone and Joint Diseases, Shanghai Institute of Traumatology and Orthopaedics, Ruijin Hospital, Shanghai Jiao Tong University School of Medicine, No. 197, Ruijin 2^nd^ Rd, Shanghai, 200025, PR China

^c^Department of Geriatrics, Ruijin Hospital, Shanghai Jiao Tong University School of Medicine, 197 Ruijin 2nd Road, Shanghai, 200025, PR China.

*E-mail addresses: wl10779@rjh.com.cn (L. Wang), zj10455@rjh.com.cn (J. Zhong), ndl12353@rjh.com.cn (D. Ni), zhengtingwang@shsmu.edu.cn (Z. Wang).

^#^Xue Chen and Haiyan Guo contributed equally to this work.

**Experimental section**

**Materials.** Lipopolysaccharide (LPS) was obtained from MedChemExpress. N-(3-dimethylaminopropyl)-N′-ethylcarbodiimide hydrochloride (EDC) and N-hydroxysuccinimide (NHS) were from TCI Development Company. Phosphate buffered solution (PBS), Dulbecco's Modified Eagle Medium (DMEM), penicillin-streptomycin and fetal bovine serum (FBS) were from Adamas Life. MRS broth and agar was obtained from Qingdao Hi-tech Industrial Park Hope Bio-technology Company. Cell counting kit-8 (CCK-8), EDTA (0.5 M pH 8.0), DCFH-DA kit and hydrogen peroxide assay kit were achieved from Beyotime. Simulated intestinal fluid (SIF) and simulated gastric fluid (SGF) were purchased from Shanghai yuanye Bio-Tech Company. Cytokine (TNF-α, IL-1β, iNOS, Arg-1, MUC2, ZO-1, Occludin) primers were obtained from Sangon. IntestiCult™ Organoid Growth Medium (Mouse) and Dulbecco's Phosphate-Buffered Saline without Ca^2+^ and Mg^2+^ (DPBS) were purchased from STEMCELL and Matrigel was obtained from CORNING. Dextran sulfate sodium salt (DSS, MW of 36000−50000 Da) and Hieff qPCR SYBR Green Master Mix was from Yeasen. Urea, citric acid, trizol reagent, NaOH, CaCl_2_, MgSO_4_, KH_2_PO_4_, NaHCO_3_, tryptone, yeast, cellobiose, and cysteine hydrochloride were obtained from Adamas. All other reagents used in this study were analytical grade.

**Strain and growth condition.** The LGG were cultured in MRS broth with shaking at 200 rpm 37 ℃ overnight to the logarithmic phase. Then LGG were subjected to centrifugation at 4000 g for 5 minutes and washed by PBS twice for the subsequent experiments. MRS agar plates were used to culture LGG cells for bacterial counts.

**Cells and growth condition.** Caco-2 cells (RRID: CVCL_0025) and RAW 264.7 cells (RRID: CVCL_0493) were obtained from the American Type Culture Collection (ATCC) and were confirmed to be contamination free before use. Both caco-2 and RAW 264.7 cells were cultured in DMEM with penicillin-streptomycin (1%) and FBS (10%). An incubator at 37 ℃ with a humid atmosphere containing 5% CO_2_ was used for cell incubation. When experiments were performed, DMEM medium without FBS and penicillin-streptomycin was used to culture cells and resuspend bacteria.

**Animals.** Male C57BL/6 mice (6-8 weeks old, 20-22 g) were obtained from Shanghai Lingchang Biotech Company (Shanghai, China). All mice were housed in a specific pathogen free (SPF) atmosphere with a 12 h light/dark cycle and constant temperature of 22 ± 2 °C. All mice were provided free fresh drinking water and standard rodent chow. All the animal experiments were approved by the Experimental Animal Ethics and Use Committee of Shanghai Jiaotong University (A2023171).

**Synthesis of Carbon dots and C dots@LGG.** C dots were fabricated with citric acid and urea through microwave pyrolysis. Briefly, 3 g citric acid and 3 g urea were fully dissolved in 10 mL ultrapure water. The solution was irradiated in a microwave oven (Midea, China) at 750 W for 6 min to obtain a brownish-black solid, which was subsequently heated in vacuum condition at 60 °C for 1 h to remove small molecules. The solid was then redispersed in ultrapure water and homogenized by ultrasound for 15 min. The new brownish-black solution was subjected to centrifugation at 3000 rpm for 30 min to remove large molecules and 10000 rpm for 20 min twice to purify the C dots solution. The resulting C dots solution was lyophilized and weighed.

EDC and NHS were used to active carboxyl groups on the C dots surface for ligation to LGG. In the reaction, the ratio of EDC and NHS was 1: 1. C dots and EDC/NHS were stirred in PBS buffer at 4 ℃ on a magnetic stirrer for 2 h and then mixed with LGG to shake at 300 rpm for another 30 min for ligation. The C dots@LGG was obtained after washing three times by PBS. The synthesized C dots@LGG were counted by plate counting method after gradient dilution with PBS.

**Characterization of C dots, LGG and C dots@LGG.** The average sizes and ζ-potentials of C dots, LGG, and C dots@LGG were measured by Zetasizer (Malvern, U.K.). For TEM observation, a drop (10 μL) of C dots solution or bacterial suspension was deposited onto an ultrathin pure carbon film and dried in the air after washed twice by ultrapure water. C dots exhibited red fluorescence under 560 nm laser excitation. LGG and C dots@LGG suspension were dropped onto a slide lined with a thin LB agar square and covered by the coverslip before observation under a confocal laser scanning microscope (CLSM, TCS SP8 STED 3X, Leica). And C dots on the surface of LGG were further confirmed using a flow cytometer (CytoFLEX LX, Beckman).

**Growth curves of LGG and C dots@LGG.** LGG and C dots@LGG were inoculated in MRS broth medium in 96-well plates. The absorbance of the medium at 600 nm was recorded at a 30 min interval for 6 h by a microplate reader (Agilent BioTek Epoch 2).

**Biocompatibility of C dots and C dots@LGG *in vitro*.**  C dots (125, 250, 500, 1000 μg/mL) and the same amount of LGG and C dots@LGG with different concentration (1 × 10 ^8^, 2 × 10 ^8^, 5 × 10 ^8^, 1 × 10 ^9^ CFU/mL) were co-incubated with caco-2 cells and RAW 264.7 cells respectively for 4 h at 37 ℃. CCK-8 assay was employed to assess the cellular activity through the measurement of the absorbance at 450 nm.

***In vitro* H_2_O_2_-scavenging and protection effect against H_2_O_2_.** The LGG and C dots@LGG were mixed with 100 μM H_2_O_2_ and reacted for 10 min. After centrifugation, hydrogen peroxide assay kit was used to detect the amount of residual H_2_O_2_ by reading the absorbance at 560 nm by a microplate reader. Then, 200 μM H_2_O_2_-treated caco-2 cells were co-incubated with DMEM medium, C dots (250 μg/mL), LGG or C dots@LGG (equally bacterial count of 1 × 10^8^ CFU/mL) for 6 h and the intracellular reactive oxygen species (ROS) was detected by DCFH-DA kit.

To evaluate the protection effect of C dots@LGG against oxidative damage, caco-2 cells were stimulated by 800 μM H_2_O_2_ in DMEM medium and treated by different dosages of LGG or C dots@LGG (1 × 10^8^, 2 × 10^8^, 5 × 10^8^, 1 × 10^9^ CFU/mL) for 4 h. Afterwards, we used CCK-8 assay to evaluate the survival rate of caco-2 cells by measuring absorbance at 450 nm by a microplate reader. The same approach was adopted to assess the antioxidant and protective effect of C dots. Meanwhile, 600 μM H_2_O_2_-treated caco-2 cells were co-incubated with DMEM medium, C dots (250 μg/mL), LGG or C dots@LGG (equally bacterial count of 1 × 10^8^ CFU/mL) for 4 h and the cell viability were detected by Calcein/PI kit.

***In vitro* anti-inflammatory effect of C dots@LGG.** RAW 264.7 cells were co-incubated with 250 ng/mL LPS in DMEM medium and treated by C dots (250 μg/mL), LGG or C dots@LGG (equally bacterial count of 1 × 10^8^ CFU/mL) for 12 h. Afterwards, cells were collected. RNA of the cells were isolated by trizol reagent and converted into cDNA using All-in-One First-Strand Synthesis MasterMix (with dsDNase) (Teyebio, Shanghai). Nano Drop 2000 (Thermo Scientific, USA) was used for RNA detecting. Subsequently, quantitative reverse transcription PCR (qPCR) was was carried out on the LightCycler® 96 (Roche). The relative mRNA expression was determined in accordance with the comparative threshold cycle method. All expression patterns were normalized to GAPDH. The primers of genes for qPCR were listed below.

GAPDH: Forward ATGGTGAAGGTCGGTGTGAA

Reverse CCCAATACGGCCAAATCCTA

TNF-α: Forward TAGCCCACGTCGTAGCAAAC

Reverse GCAGCCTTGTCCCTTGAAGA

IL-1β: Forward TTCAGGCAGGCAGTATCACTC

Reverse GAAGGTCCACGGGAAAGACAC

iNOS: Forward GCCCAGGAGGAGAGAGAT

Reverse GCAAAGAGGACTGTGGCT

Arg-1: Forward CTCCAAGCCAAAGTCCTTAGAG

Reverse GGAGCTGTCATTAGGGACATCA

MUC2: Forward ATGCCCACCTCCTCAAAGAC

Reverse GTAGTTTCCGTTGGAACAGTGAA

ZO-1: Forward TCCCAGCTTATGAAAGGGTTGT

Reverse TGGCTCCTCTCTTGCCAACTT

Occludin: Forward CTGGATCTATGTACGGCTCACA

Reverse TCCACGTAGAGACCAGTACCT

**Pro-proliferation ability of C dots@LGG *in vitro*.** Three C57BL/6 mice were sacrificed to collect their colons for crypts extraction. Colons were opened lengthwise and cut into 0.5 cm pieces in cold PBS and then washed with cold DPBS (Ca^2+^, Mg^2+^ free) for five times. Then the tissues were digested by 5 mL of crypt chelating buffer (2 mM EDTA solution configured with DPBS, sterilized with a 0.22-μm filter) and shaken gently on ice for 30 min. After that, the tissues were transferred into 5 mL cold dissociation buffer (43.4 mM sucrose and 54.9 mM D-sorbitol configured with DPBS, sterilized with a 0.22-μm filter) and were shaken for 30 s by hand to isolate colonic crypts. Then a 100 μm cell filter was utilized to filter the solution to collect the isolated crypts. The purified crypts suspension was centrifugated at 800 rpm for 5 min at 4 °C. The crypts were then resuspended with DPBS and counted under a microscope. The counted crypts were mixed evenly with Matrigel that had been thawed in advance at 4 °C, with a concentration of 200 crypts/50 μL Matrigel. Then the crypts were inoculated into a 24-well plate at the above concentration (50 μL/well) and the plate was put into the cell incubator for 15 min to solidify the Matrigel at 37℃. Then 500 μL of organoid complete medium was added, which was configured with intesticult™ organoid growth medium, penicillin-streptomycin (1%) and Y-2763210 (μM) and the crypts were cultured in a cell incubator. The colonic organoids were stimulated by LPS (100 ng/mL) and were treated by DMED medium, C dots (250 μg/mL), LGG or C dots@LGG (equally bacterial count of 1 × 10^8^ CFU/mL). The group cultured without LPS and any treatment was regarded as control group. They were co-cultured for 5 days and pictures were taken every day by a microscope. ImageJ software (NIH, Bethesda, MD, USA) was employed to work out the grown area of the colonic organoids.

Wound healing assay was performed as follows. Briefly, caco-2 cells were seeded (10^6^ cells/well) and cultured until they grew to 80% confluence. Straight scratches were made by a 200 μL pipet tip and removed the floating cells by washing them with PBS twice. Then the cells were stimulated with H_2_O_2_ (250 μM) and treated with C dots (250 μg/mL), LGG or C dots@LGG (equally bacterial count of 1 × 10^8^ CFU/mL) for 24 h. The images of the scratches were taken by a microscope at different time points (0 h and 24 h). ImageJ software was employed to calculate the healing rate of the gap.

**Analysis of the *in vitro* resistance of C dots@LGG.** Equal amounts of LGG or C dots@LGG (10^9^ CFU/mL) were diluted into 1 mL SGF (pH 2.5), respectively. The suspension was gently shaken at 37 °C for 30 min and 100 μL mixture was taken out at different time points for a colony count after a gradient dilution. The resistance to SIF (pH 6.8) and SICF (pH 7.8 with 200 μM H_2_O_2_)^[1]^ were evaluated by similar method while the co-incubated time was 2 h.

To evaluate the resistance ability of C dots@LGG in a simulated inflammatory intestinal microecological environment, we collected feces from ten healthy 8-week-old C57BL/6 mice. After adding cold 0.9% NaCl solution, mechanical homogenization (150 rpm, 5 min) was performed. Then, centrifugation was carried out five times at 100 g for 5 min and twice at 800 g for 5 min to remove non-bacterial parts. Finally, the collected supernatant was centrifugated at 4000 g for 10 min to obtain the mouse gut bacteria. The gut bacteria suspension was adjusted to 1×10^9^ CFU/mL and frozen at -80°C with 30% glycerol for subsequent experiments. The culture medium for intestinal flora is configured according to the previous scheme with a minor modified.^[2]^ Then, 8 mg CaCl_2_, 100 mg MgSO_4_, 24 mg KH_2_PO_4_, and 400 mg NaHCO_3_ were diluted with 1 L deionized water, to which 10 g yeast extract, 20 g tryptone, 1 g cellobiose, and 0.5 g cysteine hydrochloride were added.

The extracted gut bacteria (1 × 10^9^ cells) were inoculated into the nutrition medium (25 mL) with 200 μM H_2_O_2_ to mimic inflammatory circumstance, and then inoculated LGG or C dots@LGG (equally bacterial content of 2 × 10^8^ CFU) and co-cultured for 3 days in the anaerobic condition. After that, the samples were subjected to centrifugation at 1000 rpm for 5 min, followed with another centrifugation at 12000 rpm for 5 min at 4 °C. About 1 mL fermentation liquid was extracted with 2 mL anhydrous ether for 20 min. The supernatant after centrifugation at 3000 rpm 5min was extracted by 500 μL 1 M NaOH for another 20 min. Subsequently, we took the lower aqueous phase after centrifugation and then added 100 μL concentrated hydrochloric acid. Samples were transferred to a vial prior for HPLC analysis of lactic acid after filtered by 0.22 μm membranes.

**Biocompatibility of C dots@LGG *in vivo*.** C57BL/6 mice with colitis induced by 4% DSS were gavaged with PBS, C dots (8 mg/kg body weight), LGG or C dots@LGG (bacterial dose of 1 × 10^9^ CFU/mL) for 7 days. The mice were free to fresh water without DSS and gavaged with solvent as control group. On day 8, all the mice were euthanized and the main organs were taken out for histological analysis (H&E staining) to detect the biocompatibility of different treatments.

**Retention** **capacity of C dots@LGG in the intestine.** Cy7 was used to label LGG before they were modified by C dots. C57BL/6 mice were given drinking water with 4% DSS for 7 days and gavaged with Cy7-labeled LGG or Cy7-labeled C dots@LGG (equally bacterial content of 5×10^8^ CFU/mL). *In vivo* imaging system (IVIS, PerkinElmer) was employed to take fluorescent images of mice 4 h, 6 h, 8 h after gavage. Then their intestines were collected for fluorescent images acquisition at 8 h and the fluorescence intensity was quantified after that.

**Adhesion and colonization capacity of C dots@LGG.** For *in vitro* adhesion experiment, caco-2 cells were cultured in a 24-well plate until they grew to 80% confluence. After being washed twice with sterile PBS, approximately 10^7^ CFU of LGG or C dots@LGG in DMEM medium were added to the cells and co-cultured for 3 h. Afterwards, to remove loosely adherent probiotics, cells were washed three times. Then 0.1% Triton X-100 was utilized to lyse the cells and the supernatants in the wells were collected. After gradient dilution, the bacteria were cultured on MRS agar plates for 48 h for counting.

To evaluate the colonization ability of C dots@LGG, C57BL/6 mice were given drinking water with 4% DSS for one week to induce colitis. After fasting for 12 h, LGG or C dots@LGG (equal bacterial content of 1 × 10^9^ CFU/mL) were given by gavage. Mice were sacrificed 24 h post-gavage and their proximal colon tissues, distal colon tissues, cecal contents and feces were collected. The above substances were mechanically homogenized in 1 mL sterile PBS, then gradient was diluted and the amounts of bacteria were counted by standard plate count method.

**Synergistic alleviation effect of C dots@LGG on DSS-induced colitis.** After acclimation, male C57BL/6 mice were randomly divided into five groups. The healthy control group was fed with normal water and food during the experimental period, while the other four groups were fed with drinking water containing 4% DSS for one week to establish colitis. The healthy control and DSS model group were only gavaged with 200 μL of PBS, while the C dots, LGG, and C dots@LGG groups were orally gavaged with equal volume of C dots solution (8 mg/kg body weight) and bacterial suspensions (bacteria dose, 1 × 10^9^ CFU/mL) daily for one week. Their body weight and DAI score were recorded and their feces were gathered every day. The colon lengths were measured and colon pictures were taken on day 9. Distal colons of 1 cm were reserved for histological analysis. The remaining colon tissues and the previously collected feces were frozen at -80°C for subsequent experiments. The TNF-α, IL-1β expressions were examined by qPCR. The content of myeloperoxidase (MPO) of colon tissues were detected by immunohistochemistry and the contents of malondialdehyde (MDA), superoxide dismutase (SOD) and catalase (CAT) were detected by MDA kit, SOD kit and CAT kit, respectively.

***In vivo* intestinal barrier repair and pro-proliferative capacity.** The MUC2, ZO-1 and Occludin expressions were examined by qPCR according to the aforementioned protocol. For immunofluorescence staining analysis, paraffin sections were dewaxed, antigen repaired, quenched for autofluorescence, and blocked for immunofluorescence staining. The sections were stained by anti-ZO-1 antibody (1: 100) and anti-Occludin antibody (1: 200). The nuclei was stained by DAPI. Finally, they were observed and images were collected with a fluorescence microscope (Leica).

For Alcian blue-Periodic acid Schiff (AB-PAS) staining, the paraffin colon tissue sections were successively stained with Alcian blue staining solution and Schiff Reagent staining solution for 20 min, respectively, after dewaxed and rehydration. Then, it was rinsed with running water for 10 min. Subsequently, the nuclei were stained with hematoxylin. After differentiation with acidic differentiating solution and bluing with Scott's bluing solution, dehydration and mounting were performed. The stained sections were observed and images were collected with a microscope.

For immunohistochemical analysis, paraffin slides were dewaxed, rehydration, antigen repaired, endogenous peroxidase blocked, and serum blocked before staining of Ki-67. Sections were incubated with primary antibody overnight at 4 °C. After PBS washing, secondary antibody incubation was done for 50 min at room temperature. Then, after another wash, DAB chromogenic solution was added and rinsed with running water to stop the reaction. Subsequently, nuclei were stained with hematoxylin. Finally, sections were observed under a microscope and images were collected.

ImageJ software was used to analyze the relative density of ZO-1, Occludin, AB-PAS and Ki-67.

**Bacterial Diversity Analysis.** Fecal samples were frozen at -80 °C for the subsequent 16s rDNA sequencing analysis. Briefly, microbial genomic DNA was extracted by the CTAB method and amplified by PCR. The amplified PCR products were purified and used to prepare sequencing libraries. Then, the libraries were sequenced on an Illumina NovaSeq platform. At last, the obtained sequencing data were analyzed to determine the microbial community composition.

**Statistical analysis.** Statistical analysis was performed using GraphPad Prism 9.5, and data were presented as mean ± SD. Unpaired student’s *t* test and one-way ANOVA were used in statistical significance analysis. **p* < 0.05, ***p* < 0.01, ****p* < 0.001, and *****p* < 0.0001 represented different statistical significances. ns means not significant.

**Reference**

[1] F. Cao, L. Jin, Y. Gao, Y. Ding, H. Wen, Z. Qian, C. Zhang, L. Hong, H. Yang, J. Zhang, Z. Tong, W. Wang, X. Chen, Z. Mao, *Nat Nanotechnol* **2023**, 18, 617.

[2] X. Cui, X. Wang, X. Chang, L. Bao, J. Wu, Z. Tan, J. Chen, J. Li, X. Gao, P. C. Ke, C. Chen, *Proc Natl Acad Sci U S A* **2023**, 120, e2218739120.

**
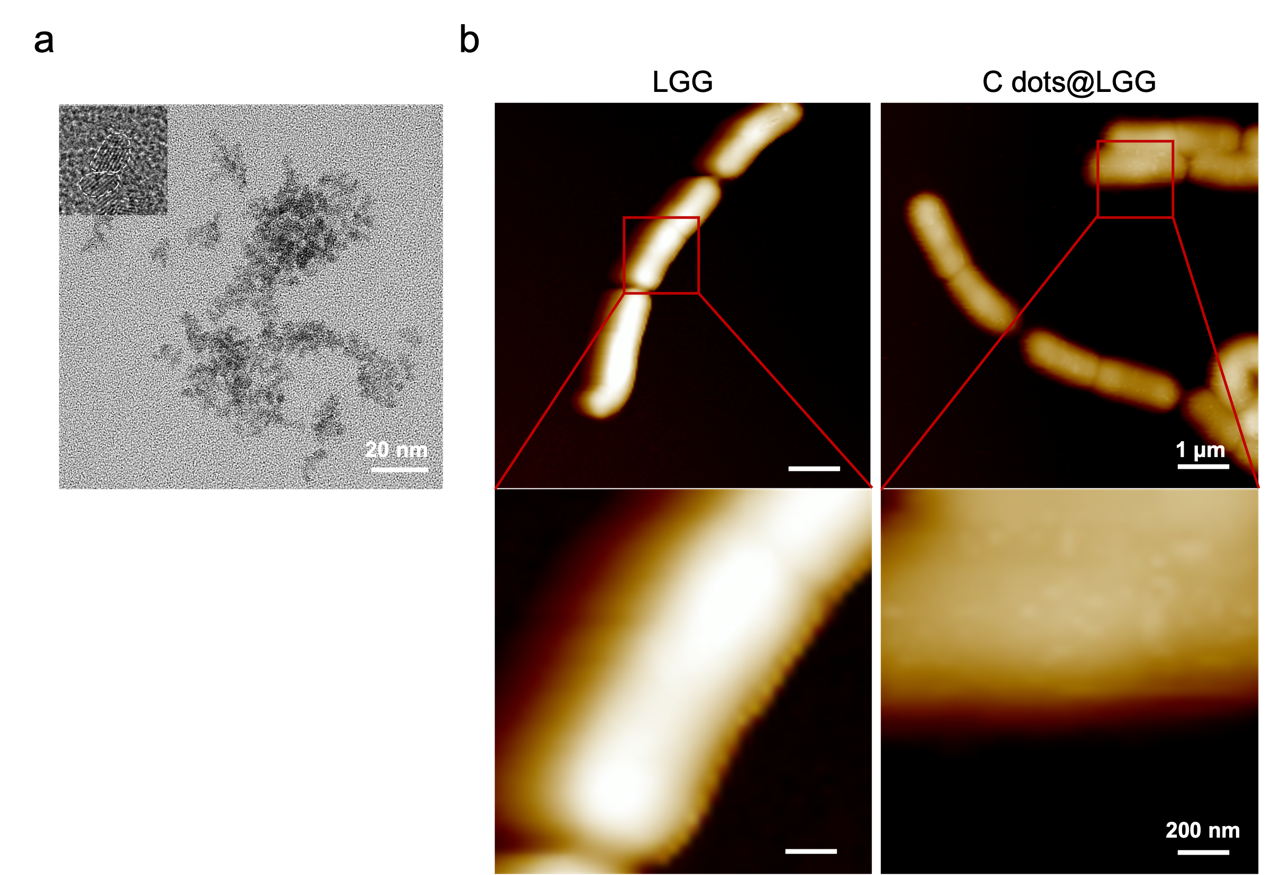
**

**Figure S1.** Characterization of C dots particles. (a) TEM image of C dots. Scale bar: 20 nm. (b) AFM images of bare LGG and C dots@LGG. Scale bar: 1 μm (above) and 200 nm (below).

**
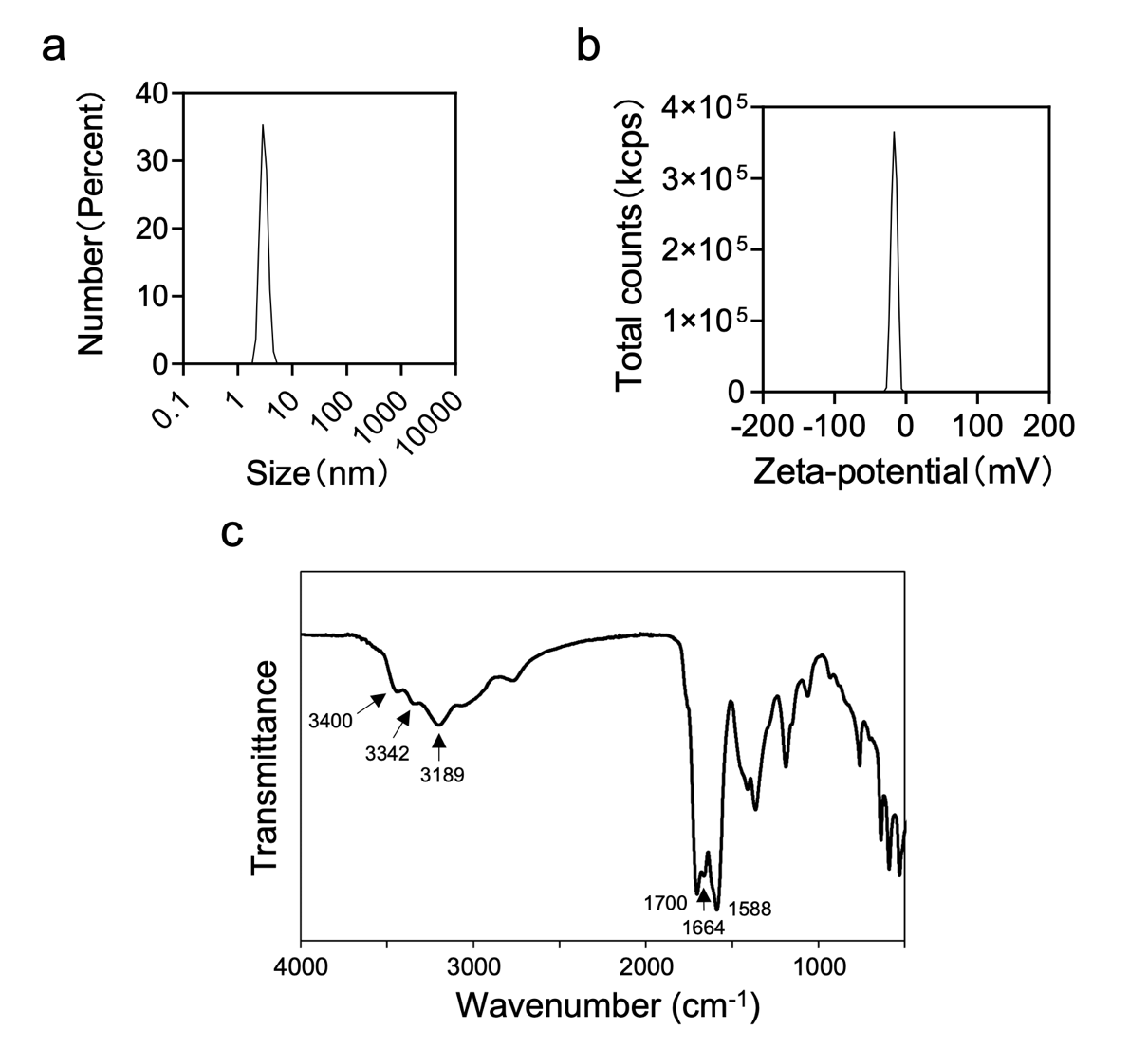
**

**Figure S2.** Characterization of C dots particles. The particle size (a) and zeta potential (b) of C dots were measured by DLS. (c) FT-IR spectrum of C dots. The absorbance bands at 3189, 3342 and 3400 cm−1 were attributing to the N-H and O-H stretching vibrations, respectively. The peaks at 1700, and 1664, 1588 cm−1 were attributed to the stretching vibration of C = O (ketone, carboxyl, or amide groups) and C = C/C = N, respectively.


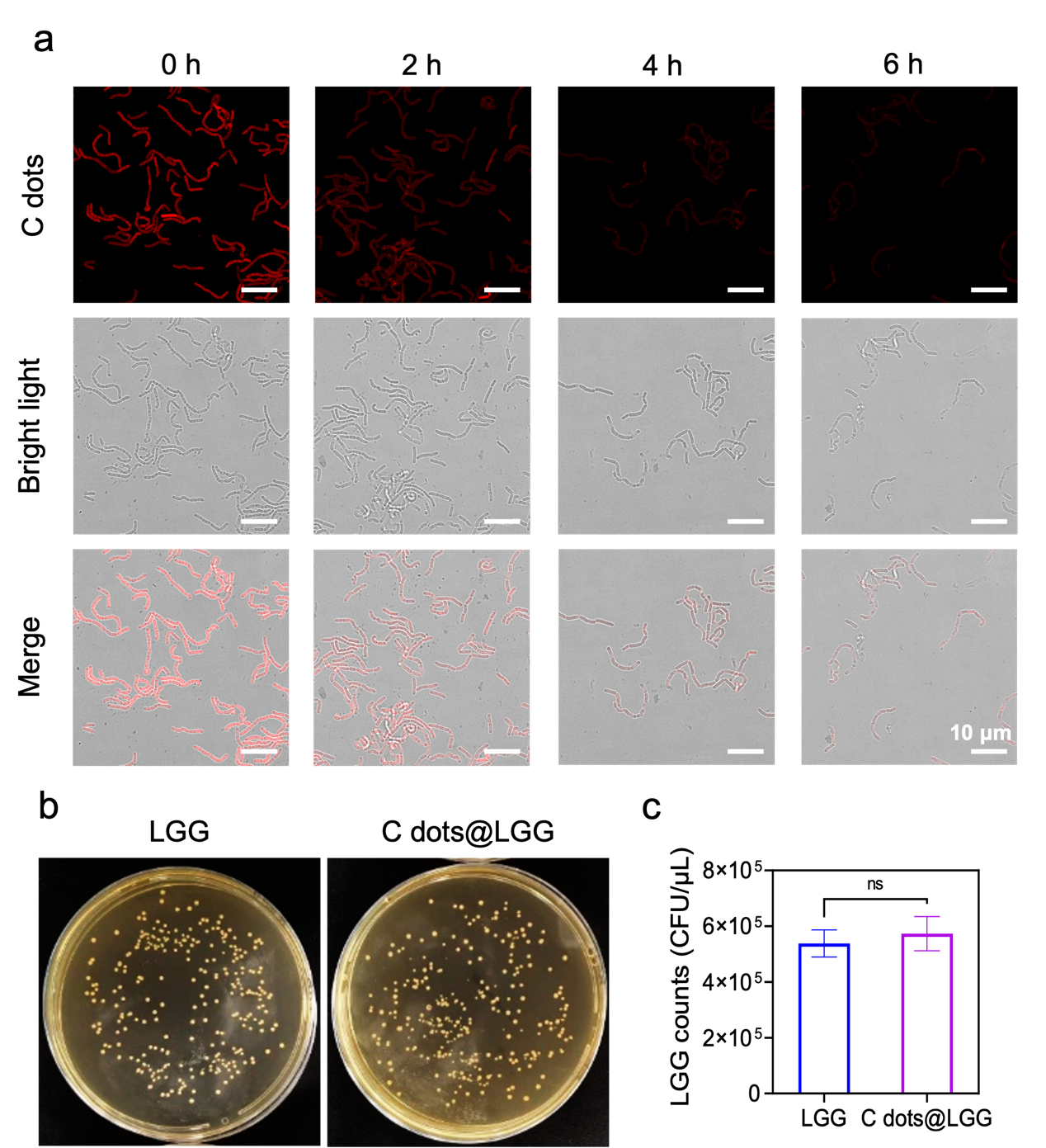


**Figure S3.** (a) CLSM images of C dots@LGG after being cultured in MRS medium for different time periods. Scale bar: 10 μm. (b) Growth condition of LGG and C dots@LGG. (c) Survival of LGG modified by C dots (n = 3). ns stands for not significant.

**
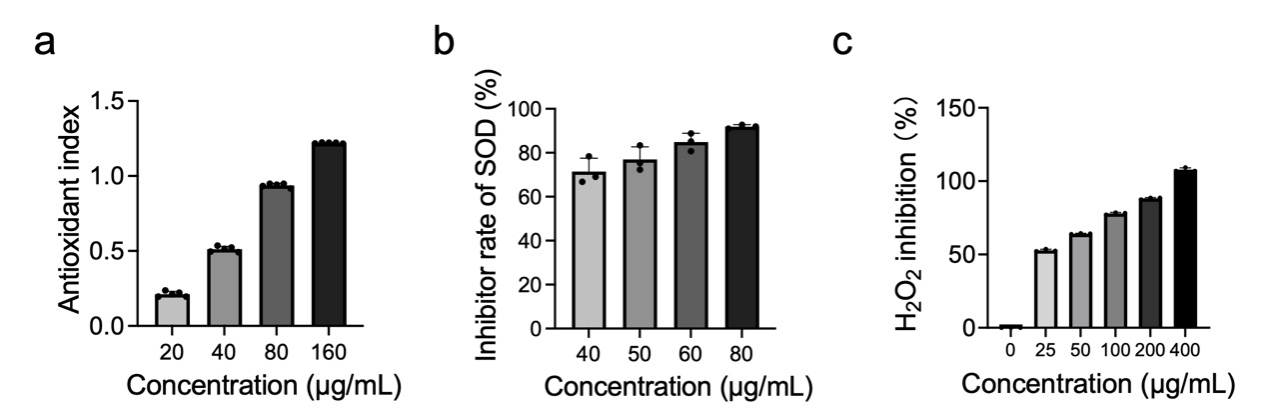
**

**Figure S4.** ROS scavenging capacity of C dots. (a) Total antioxidant ability of C dots. (b) SOD enzyme activity of C-dots at different concentrations. (c) The H_2_O_2_ scavenging capacity of C dots.


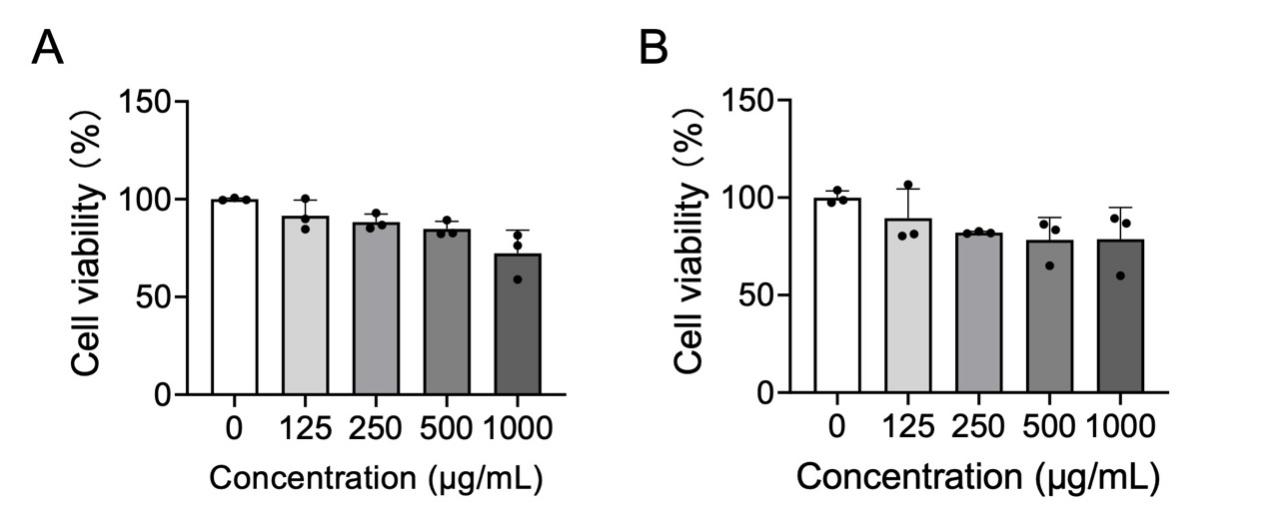


**Figure S5.** Biocompatibility of C dots. Survival rate of caco-2 cells (a) and Raw 264.7 cells (b) after co-incubated with C dots for 4 h (125, 250, 500, 1000 μg/mL). Values were mean ± SD (n = 3).


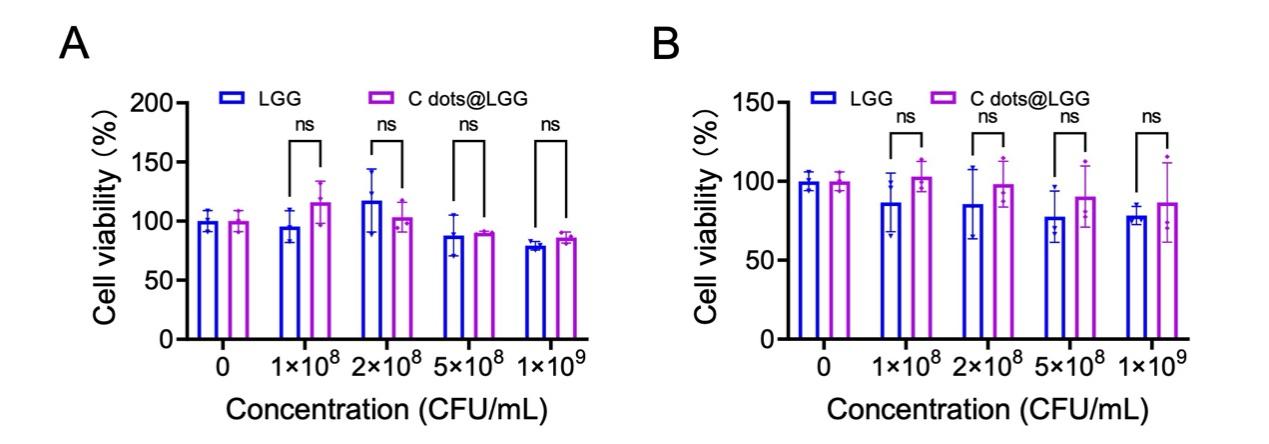


**Figure S6.** Biocompatibility of LGG and C dots@LGG. Survival rate of caco-2 (a) and Raw 264.7 cells (b) after treatments with LGG and C dots@LGG (with equal bacteria counts of 1 × 10^8^, 2 × 10^8^, 5 × 10^8^, 1 × 10^9^ CFU/mL). Values were mean ± SD (n = 3). Statistical analysis was carried out by Student’s t test. ns stands for not significant.

**
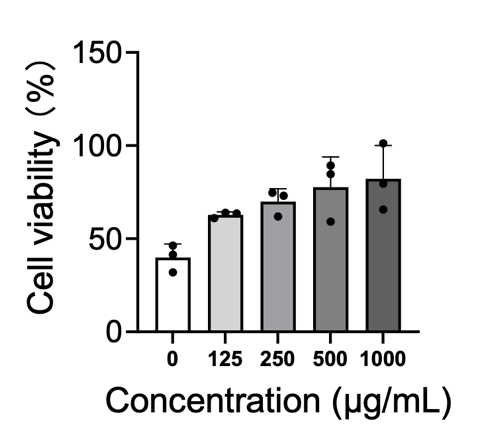
**

**Figure S7.** Protective effect of C dots for caco-2 cells against 800 μM H_2_O_2_. Values were mean ± SD (n = 3)


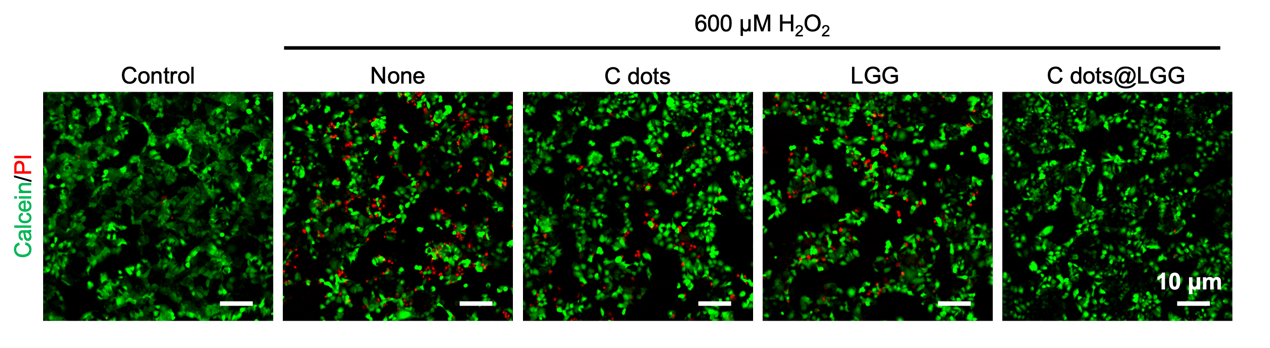


**Figure S8.** Protective effect of C dots@LGG for caco-2 cells against 600 μM H_2_O_2_. Calcein/PI fluorescence images of H_2_O_2_-treated caco-2 cells after different treatments. Scale bar: 10 μm.


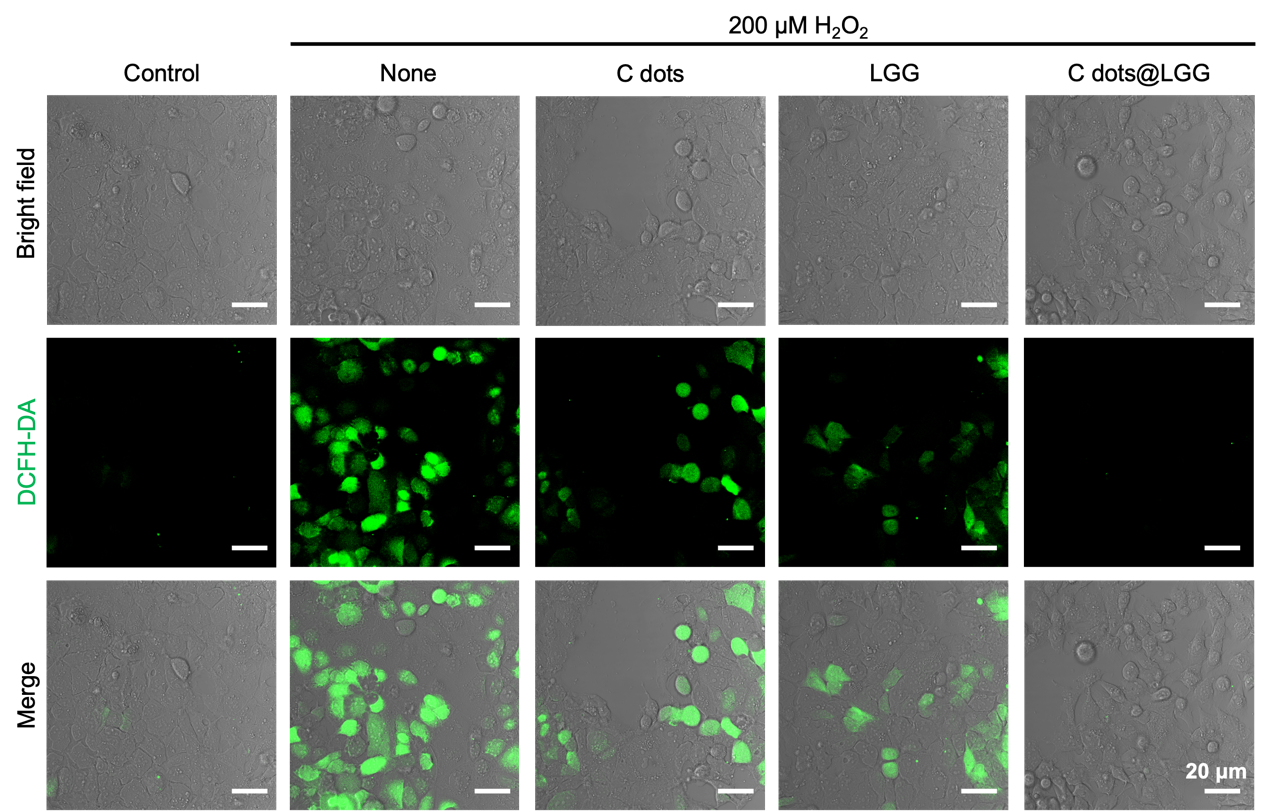


**Figure S9.** Intracellular ROS levels of H_2_O_2_-treated caco-2 cells after different treatments. Scale bar: 20 μm.


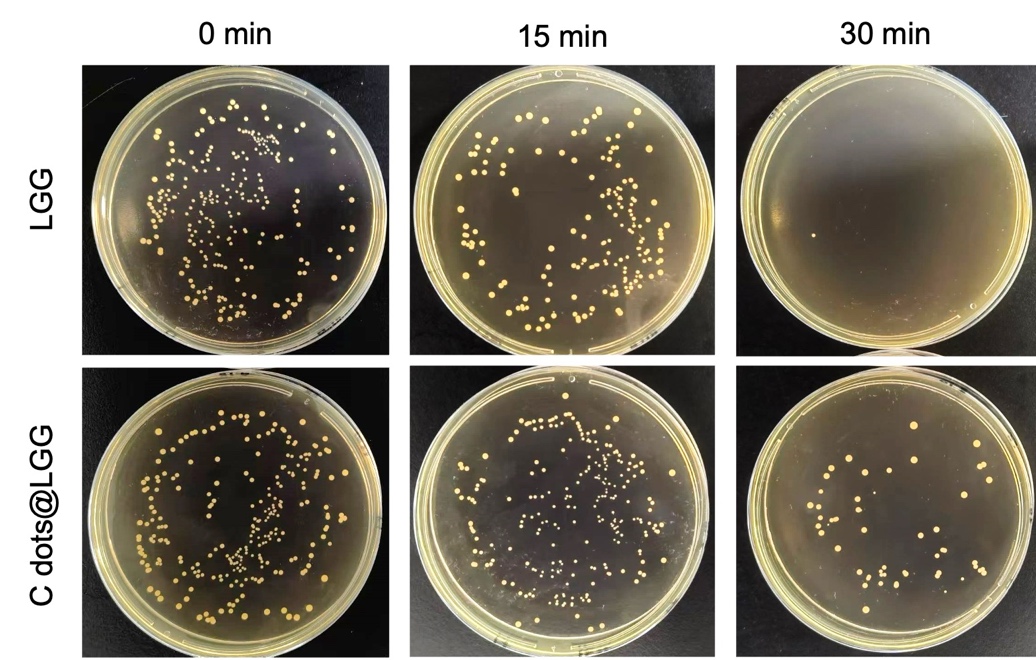


**Figure S10.** The representative images of spread plates of LGG and C dots@LGG after incubated in SGF with pH 2.5.


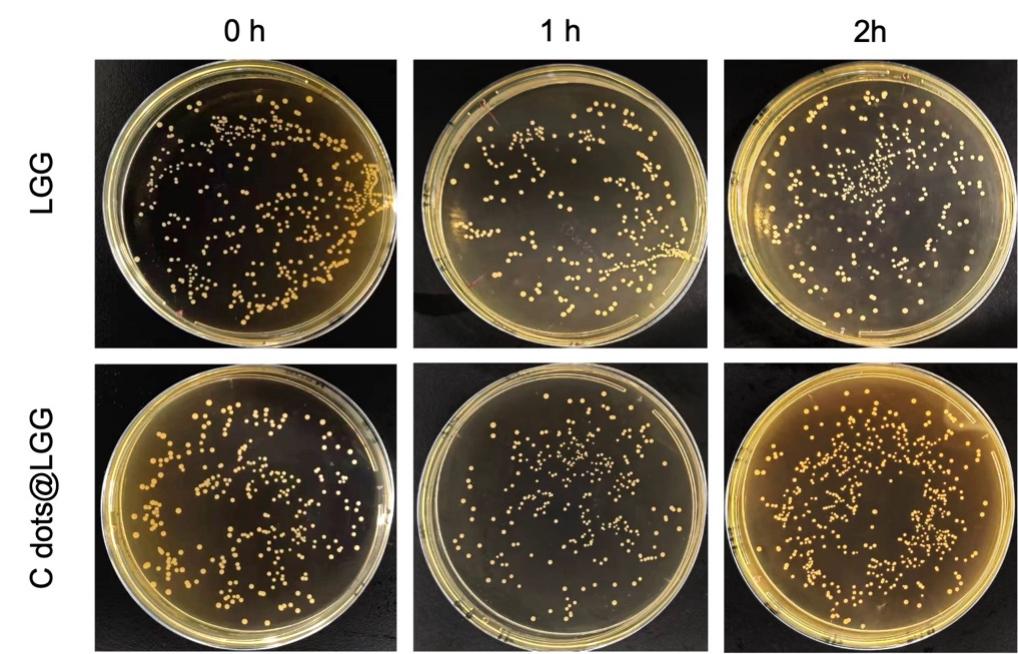


**Figure S11.** The representative images of spread plates of LGG and C dots@LGG after incubated in SIF.


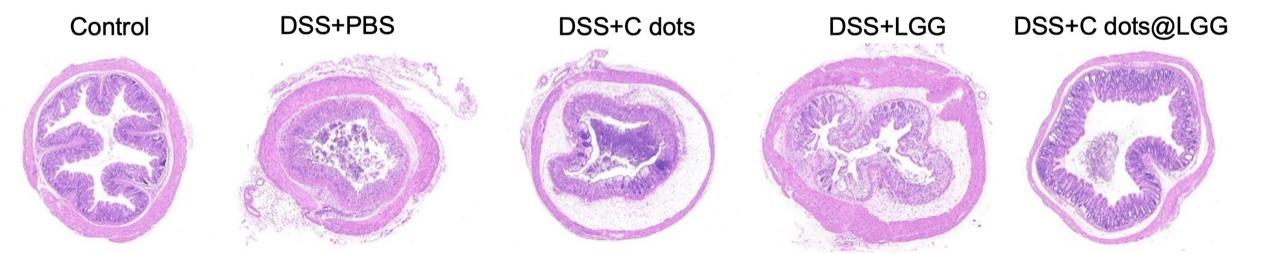


**Figure S12.** Representative histological pictures of the distal colon of healthy control group, model group, C dots group, LGG group and C dots@LGG group.


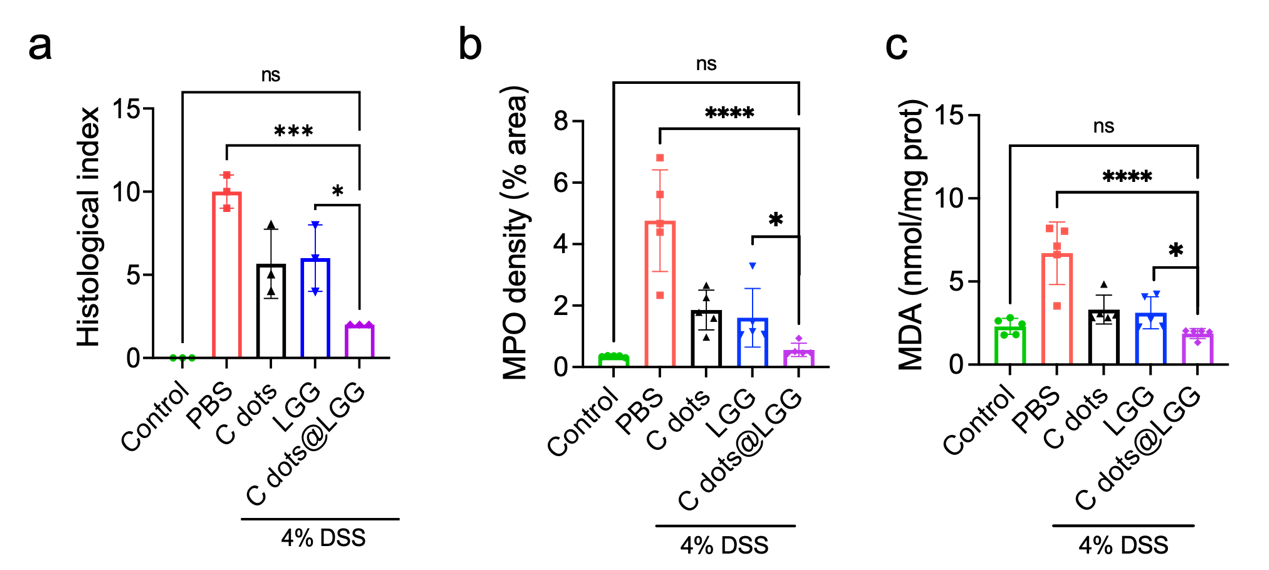


**Figure S13.** (a) Histological index of distal colon tissues from mice in control, DSS model, C dots, LGG and C dots@LGG group on day 9 (n = 3). (b) Mean density of MPO in colon sections calculated by ImageJ software (n = 5). (c) Contents of MDA of colon tissues from mice in different groups (n = 5). Values were mean ± SD. Statistical analysis was carried out by means of one-way ANOVA. **p* < 0.05, ****p* < 0.001 and *****p* < 0.0001. ns stands for not significant.


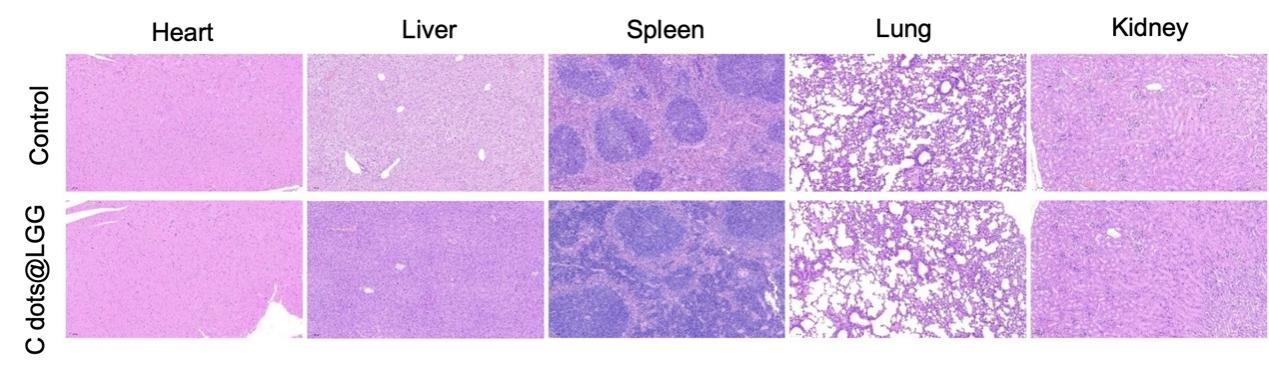


**Figure S14**. Biosafety of C dots@LGG *in vivo*.
